# Chain-Shattering Polymers as Degradable Microdispersive Solid-Phase Extraction Sorbents

**DOI:** 10.1021/acs.analchem.2c01301

**Published:** 2022-06-13

**Authors:** Cecilia Ortega-Zamora, Javier González-Sálamo, Marcelle D. Perretti, David Santana, Romen Carrillo, Javier Hernández-Borges

**Affiliations:** † Departamento de Química, Unidad Departamental de Química Analítica, Facultad de Ciencias, Universidad de La Laguna (ULL), Avda. Astrofísico Fco. Sánchez, s/n, 38206 San Cristóbal de La Laguna, Spain; ‡ Instituto Universitario de Enfermedades Tropicales y Salud Pública de Canarias, Universidad de La Laguna (ULL), Avda. Astrofísico Fco. Sánchez, s/n, 38206 San Cristóbal de La Laguna, Spain; § Department of Chemistry, Sapienza University, P.le Aldo Moro, 5, 00185 Rome, Italy; ∥ Instituto de Productos Naturales y Agrobiología, CSIC, Avda. Astrofísico Fco. Sánchez, s/n, 38206 La Laguna, Spain

## Abstract

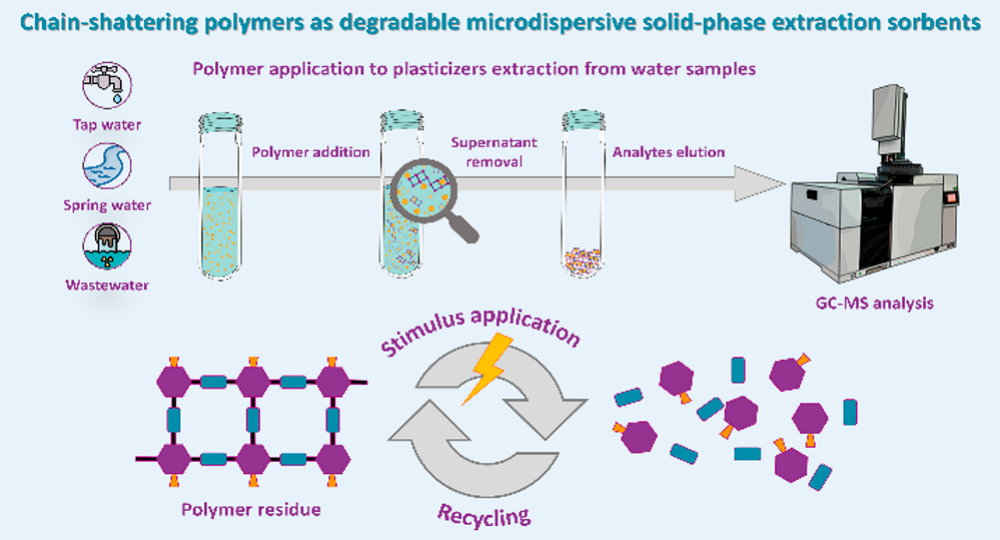

A chain-shattering polymer (CSP) has been proposed as a microdispersive solid-phase extraction (μdSPE) sorbent in a proof-of-concept study of degradable materials for analytical purposes. The responsive CSP was synthesized from 1,3,5-tris(bromomethyl)-2-nitrobenzene acting as the self-immolative trigger responsive unit and 2,6-naphthalenedicarboxylic acid as aromatic linker to enhance noncovalent aromatic interactions with the analytes. The CSP was characterized and applied as a μdSPE sorbent of a group of plasticizers, which were selected as model analytes, from different types of environmental water samples (tap, waste, and spring waters). Gas chromatography coupled to mass spectrometry detection was used for analyte determination. Mean recovery values were in the range of 80%–118% with RSD values below 22%. After the extraction, the polymer could be efficiently degraded by UV irradiation or by chemical reduction, recovering the aromatic linker. This work has proved the potential of CSPs as recyclable sorbents, paving the way to more environmentally benign analytical procedures.

The use of polymers in the analytical chemistry field has become essential in the development and application of a high number of analytical methodologies. In particular, they have been widely used as extraction sorbents under different formats: in bulk, packed in cartridges or columns, forming thin films, covering nanoparticles, as part of nanocomposites, and more.^1,2^ Once used, such polymeric sorbents are commonly discarded without really paying attention to their final destinations, although in very few cases they are reused several times, with the subsequent risk of analyte carry over or decrease in extraction efficiency.^3^

Concerning the introduction of new extraction sorbents, which is clearly one of the most important trends in this field,^4,5^ the applications of stimuli responsive materials have awakened special attention in the last years.^6^ Such smart/intelligent materials are able to undergo changes in solubility, volume, and/or conformation, among others, in response to an external stimulus which can be either biological, chemical, or physical.^7−9^ Interestingly, responsiveness of materials could also be employed to enhance their environmental virtues. Indeed, responsive polymers have found to be an excellent alternative to traditional recyclable materials.^10^ Polymer recyclability is critical to sustainability efforts worldwide, and as a consequence, greener materials are demanded in order to meet the increasing social and legal standards.^11,12^ Such a renewed interest in degradable polymers has led to an extensive search for new mechanisms to breakdown polymers. Those responsive and degradable materials must be stable under ambient conditions, which allows the polymer to perform the task it was designed for. However, under a specific stimulus, the polymer is degraded.

In this regard, self-immolative polymers (SIPs) are particularly interesting because one triggering event is able to disassemble the polymer spontaneously through a domino-like fragmentation pattern.^13^ Traditionally, only those polymers which disassemble through a chain end-initiated degradation, from head to tail, are called SIPs.^14^ However, SIPs may be designed to undergo side-chain-initiated self-immolation reactions, although they are named chain-shattering polymers (CSPs). Such materials are able to spontaneously degrade along the main chain with a triggering event occurring at each of the monomer units.^15,16^ CSPs display some advantages with respect to SIPs. Particularly, they are easier to synthesize, and they achieve faster degradation rates than end-capped SIPs due to the higher concentrations of potential cleavage sites.^17^ Both SIPs and CSPs base their functionality on self-immolative units. The most basic and commonly used self-immolative units are phenols^18^ or anilines^19^ with a good leaving group (LG) linked to a methylene (i.e., carboxylates, phenols, and carbamates) in *ortho* or *para* positions (Scheme 1). In those units, a quinone-methide elimination can take place, releasing the LG and degrading the system upon the application of the right stimulus. In the specific case of the anilines (Scheme 1a), for example, if the amine is suitably masked, the system is stable, but with the correct stimulus, which can be the addition of an enzyme, an acid, or a reducing agent, the amino group is unmasked. Then, the system becomes unstable. A spontaneous electronic cascade yields a 1,6-elimination that releases the LG and concomitantly generates azametilenquinone that normally traps a nucleophile from the environment, for example, water, giving 4-aminobenzyl alcohol. Interestingly, a triple self-immolation process can occur in both *ortho* and *para* positions, which can be intelligently used to make degradable complex structures and materials (Scheme 1b).^20^ Multiple self-immolation is particularly important for CSPs, because it is the basis of double or triple self-immolative nodes that allows one to build up the polymer.

**Scheme 1:**
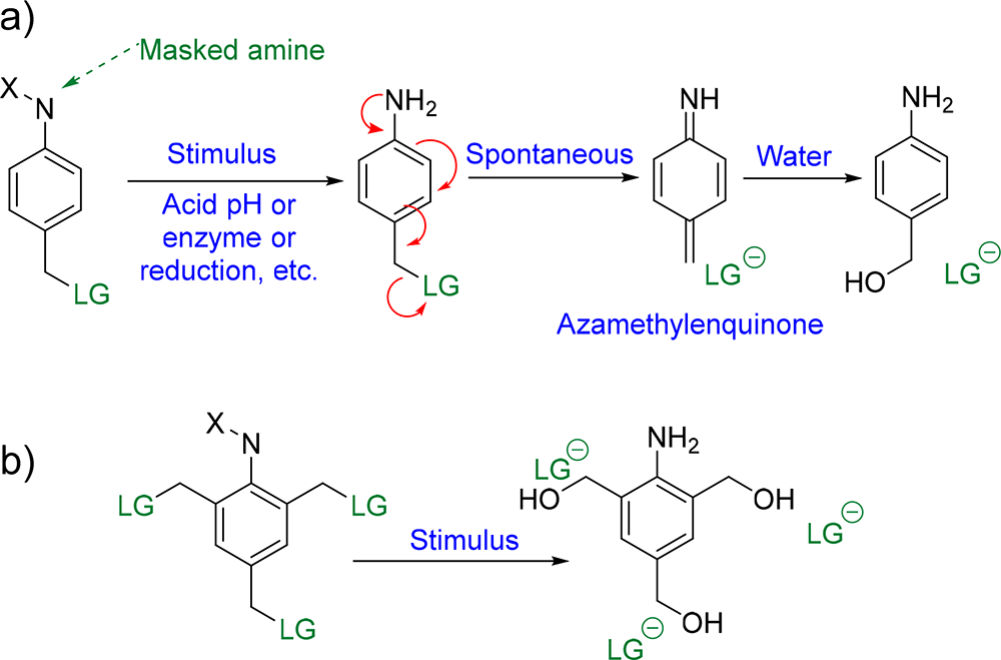
(a) Self-Immolative Unit with a Masked Amine. (b) Triple Self-Immolative Unit

As a result of their efficient disassembly chemistry, both SIPs and CSPs have found a wide range of applications from signal amplification to drug delivery.^13−15,20^ However, to the best of our knowledge, and despite their potential use as degradable sorbents in the analytical chemistry field, none of them have been explored in this field yet.

Herein, we have proved that SIPs could be used as extraction sorbents in microdispersive solid-phase extraction (μdSPE) and could be later disassembled once the extraction has taken place (Figure 1). Considering the aim of our work, we chose to employ a CSP as they are easier to synthesize, and they are degraded faster than SIPs. Since μdSPE is a highly advantageous extraction technique as a result of its simplicity, rapidity, and low consumption of sorbents and reagents, the applications of a CSP as a μdSPE sorbent and, in general, to any SPE procedure could add an additional value from an environmental point of view. For this proof-of-concept application, we have selected as model analytes a group of phthalic acid esters (PAEs) and an adipate because they are one of the main types of plasticizers used in the plastic industry, even when they produce several endocrine system disorders in humans,^21^ which has forced their restriction by governmental agencies worldwide.^22,23^

**Figure 1:**
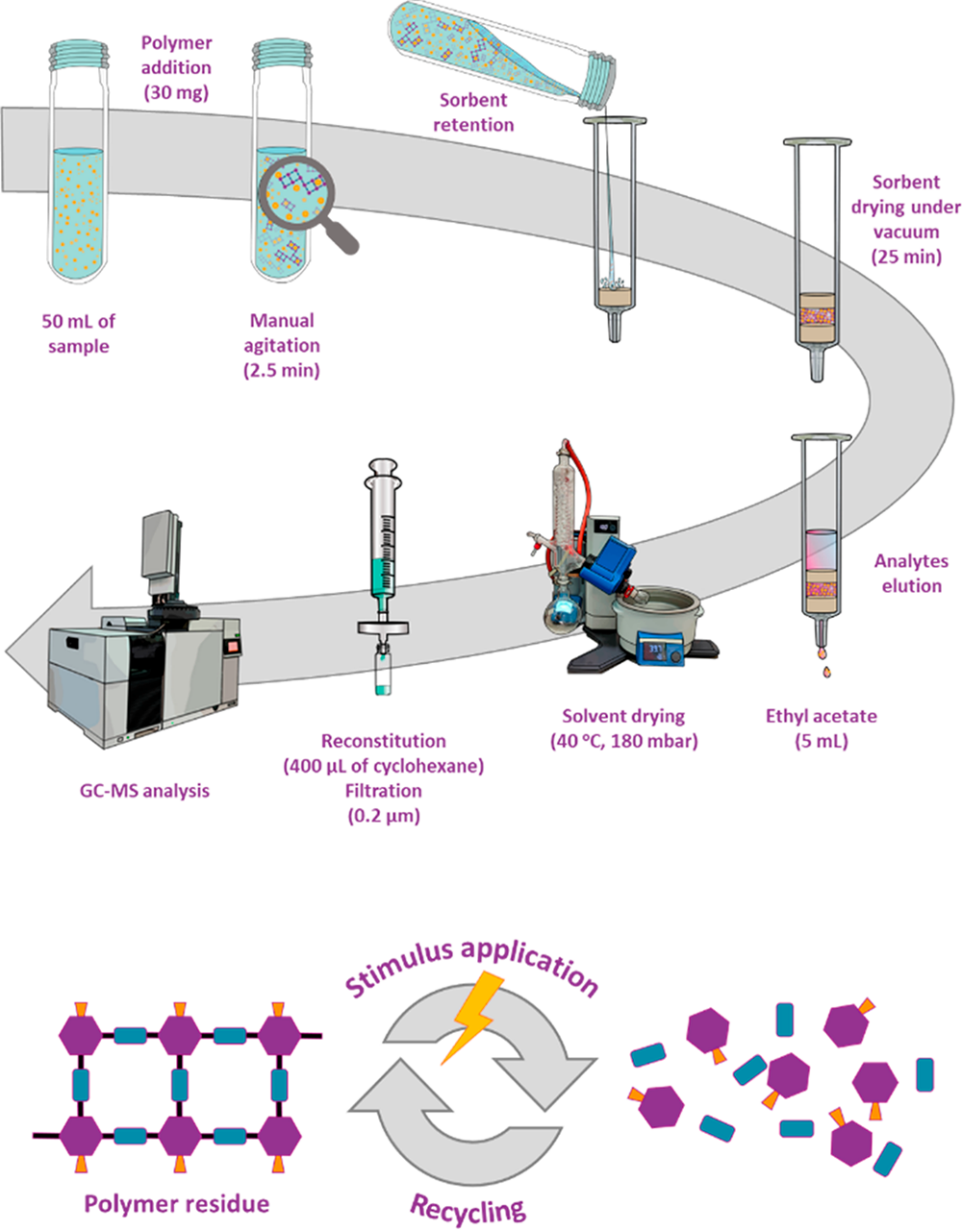
Schematic representation of the optimum μdSPE procedure applied in this work and the efficient degradation/recycling of the polymer after its use.

## Experimental Section

### Standards and Solutions

Di-*n*-pentyl phthalate (DNPP, CAS 131-18-0), benzyl butyl phthalate (BBP, CAS 85-68-7), and di(2-ethylhexyl) adipate (DEHA, CAS 103-23-1) from Dr. Ehrenstorfer (Augsburg, Germany) and diisopentyl phthalate (DIPP, CAS 605-50-5), dihexyl phthalate (DHP, CAS 84-75-3), dicyclohexyl phthalate (DCHP, CAS 84-61-7), di(2-ethylhexyl) phthalate (DEHP, CAS 117-81-7), di-*n*-octyl phthalate (DNOP, CAS 117-84-0), diisononyl phthalate (DINP, CAS 20548-62-3), and diisodecyl phthalate (DIDP, CAS 89-16-7) from Sigma-Aldrich (Madrid, Spain) were used as analytical standards. Dibutyl phthalate-3,4,5,6-d_4_ (DBP-*d*_4_, CAS 93952-11-5), DNPP-3,4,5,6-d_4_ (DNPP-*d*_4_, CAS 358730-89-9), DHP-3,4,5,6-d_4_ (DHP-*d*_4_, CAS 1015854-55-3), and DEHP-3,4,5,6-d_4_ (DEHP-*d*_4_, CAS 93951-87–2) were used as internal standards (ISs). All of them had a purity higher than 97.0%. The structures and some of the chemical properties of the studied PAEs and DEHA are shown in Table S1 of the Supporting Information. Individual solutions of each analyte and IS were prepared in cyclohexane at concentrations between 900 and 1100 mg/L, from which mixed working solutions of different concentrations were prepared. All of them were stored in the dark at −18 °C. Chemicals used for the synthesis of **CSP-1** are specifically indicated in the Polymer Synthesis section.

Milli-Q water was obtained from tap water previously purified using an Elix Essential water purification system, which was then deionized using a Milli-Q gradient A10 system, both from Millipore (Burlington, MA, USA). Methanol (MeOH) and acetonitrile (ACN) of high-performance liquid chromatography–mass spectrometry (HPLC-MS) grade and cyclohexane (purity 99.7%) were from VWR International Eurolab (Barcelona, Spain), and ethyl acetate (EtOAc) hypergrade for LC-MS and acetone for gas chromatography–mass spectrometry (GC-MS) were from Merck (Darmstadt, Germany).

A sulfuric acid (95%, w/w, VWR International Eurolab) solution of Nochromix from Godax Laboratories (Maryland, USA) was used to clean the volumetric glassware for 24 h, while nonvolumetric glassware was cleaned using a Muffle Carbolite CWF 11/13 from Nabertherm GmbH (Lilienthal, Germany) by heating to 550 °C for 4–5 h. In addition, all plastic material used during sample pretreatment, such as pipette tips, gloves, or filters, was free of PAEs.

### Equipment and Software

GC separation was carried out using an 8860 GC system acquired from Agilent Technologies (Santa Clara, CA, USA), provided with an autosampler and coupled to a 5977B single quadrupole MS detector, both controlled by Enhanced MassHunter software from Agilent Technologies. Separation was achieved with a HP-5ms Ultra Inert column ((5%-phenyl)-methylpolysiloxane 30 m × 250 μm × 0.25 μm) also from Agilent Technologies. Helium was used as the carrier gas at a flow rate of 1.2 mL/min. The thermal gradient program was as follows: temperature was kept at 60 °C for 1 min, then increased up to 170 °C at 40 °C/min at a rate of 10 °C/min up to 310 °C, where the temperature was held for 3 min, achieving a total run time of 20.75 min. Injection (2 μL) was carried out at 280 °C through the splitless mode, opening the split after 0.75 min from the injection, with a purge flow to a split vent of 40 mL/min. The rest of the parameters established were the following: transfer line temperature set at 280 °C, ion source temperature at 230 °C, and ionization energy at −70 eV. Analytes were detected using the single ion monitoring (SIM) mode. Table S2 of the Supporting Information shows the quantifier and the two qualifier ions as well as the retention times of the adipate, the PAEs and ISs.

The pH and conductivity measurements were carried out using a Five Easy Plus pH/mV meter from Mettler Toledo (Columbus, OH, USA) and a CM 35+ conductivity meter from Crison (Barcelona, Spain), respectively. Solvent evaporation was performed with a RV8 rotary evaporator equipped with a HB thermostatic bath and a CVC 3000 vacuum pump with a vacuum controller from VWR International Eurolab.

### Samples

Tap, waste, and spring waters were used for method validation. Tap water was collected at our laboratory (San Cristóbal de La Laguna, Tenerife. Spring water was collected in a water gallery, 5100 m inside the mountain at Guía de Isora, a municipality located in the southwest of Tenerife (Canary Islands, Spain), and waste water was collected in a waste water treatment plant situated in Valle de Guerra, also in Tenerife. In addition, three more tap water samples collected at the towns of San Cristóbal de La Laguna, Santa Cruz de Tenerife, and Tacoronte, one spring water sample collected in another gallery located at the north of Tenerife (5000 m inside the mountain), and five more waste water samples collected at different waste water treatment plants of Tenerife were analyzed. Tap and spring water samples were directly submitted to the extraction procedure, but waste water samples were previously filtered through Durapore polyvinylidene fluoride (PVDF) filter membranes with pore sizes of 0.22 μm from Merck Millipore (Burlington, MA, USA).

### Polymer Synthesis

The synthesis of polymer **CSP-1** consisted of two stages: (i) nitration reaction and (ii) polymerization. Detailed information, including NMR spectra of the intermediate products, can be found in the Supporting Information.

### Titration

Potentiometric titration was performed to determine the p*K*_a_ value of the synthesized polymer through the Gran method using a Five Easy Plus pH/mV meter previously adjusted with buffered solutions of pH 4, 7, and 10. For this purpose, 40 mg of the polymer was added to 10 mL of Milli-Q water and titrated in triplicate with NaOH 0.1 M previously standardized with potassium hydrogen phthalate (additions of aliquots of 2 μL were developed).

### Adsorption Studies

The synthesized polymer was studied as an adsorbent of BBP from Milli-Q water. Samples were prepared with 2 mg of the polymer to which a certain volume of BBP solution of 10 mg/L was added to reach initial concentrations in the range 10–325 mg/L in a total volume of 5 mL of water. The suspensions were stirred manually for 1 min and allowed to stand for 12 h to reach equilibrium. Then, the sorbent was filtered through a Chromafil Xtra PET-20/25 filter (pore size of 0.20 μm). The concentration of BBP in the filtered solution was determined using a VWR-Hitachi LaChrom Elite 20149 HPLC system equipped with a pump HTA L-2130, an autosampler L-2200, a thermostated column system L-2300, and an ultraviolet (UV) detector L-2400. BBP quantification was performed with an Eclipse Plus C_18_ column (10 cm × 4.6 mm, 3.5 μm) and an Eclipse Plus C_18_ precolumn (12.5 × 4.6 mm, 5 μm) from Agilent Technologies, using a mobile phase at a flow of 1.0 mL/min, initially composed of ACN:H_2_O 50:50 (v/v). In addition, the injection volume was 20 μL, and the detector wavelength was set at 226 nm.

### Microdispersive Solid-Phase Extraction Procedure

Here, 30 mg of **CSP-1** and 50 mL of the water sample were placed in a 50 mL volumetric flask and vigorously shaken by hand for 2.5 min (Figure 1). Then, the sorbent was retained in an empty glass column with two polytetrafluoroethylene (PTFE) frits placed at the bottom. Once all the sample was passed through the column, and the polymer retained onto the frits, the flask was washed with 2 mL of Milli-Q water to carry the rest of the sorbent left in it. This portion of the Milli-Q water was also passed through the column. Finally, the sorbent was packed placing another PTFE frit on top, and it was dried under vacuum for 25 min. Afterward, the analytes were eluted with 5 mL of EtOAc, which were collected in an Erlenmeyer flask and evaporated to dryness on a rotary evaporator at 40 °C and 180 mbar. Subsequently, residue reconstitution was carried out with 400 μL of cyclohexane. Finally, the resulting extract was filtered through a 0.2 μm PVDF filter from Whatman (GE Healthcare, USA) and injected (2 μL) in the GC-MS system.

## Results and Discussion

Current trends in sorbent-based microextraction procedures are focused on the use of extremely low amounts of sorbents, with low consumption of elution solvents as well as other reagents.^24^ The first of them includes mainly the use of nanomaterials (i.e., carbon-based, metal, and covalent organic frameworks, nanoparticles of different nature) as well as new polymers, among others, alone or combined with different materials.^24^ Among the different microextraction configurations available, μSPE is one of the most used nowadays as a result of the advantages previously mentioned.

Frequently, and despite the fact that low amounts of the previously mentioned sorbents are used in microextraction procedures, they are not initially designed in order to take into consideration their final destination after their use. Instead, they are discarded without even considering their recycling.

### Polymer Synthesis and Characterization

**Scheme 2:**
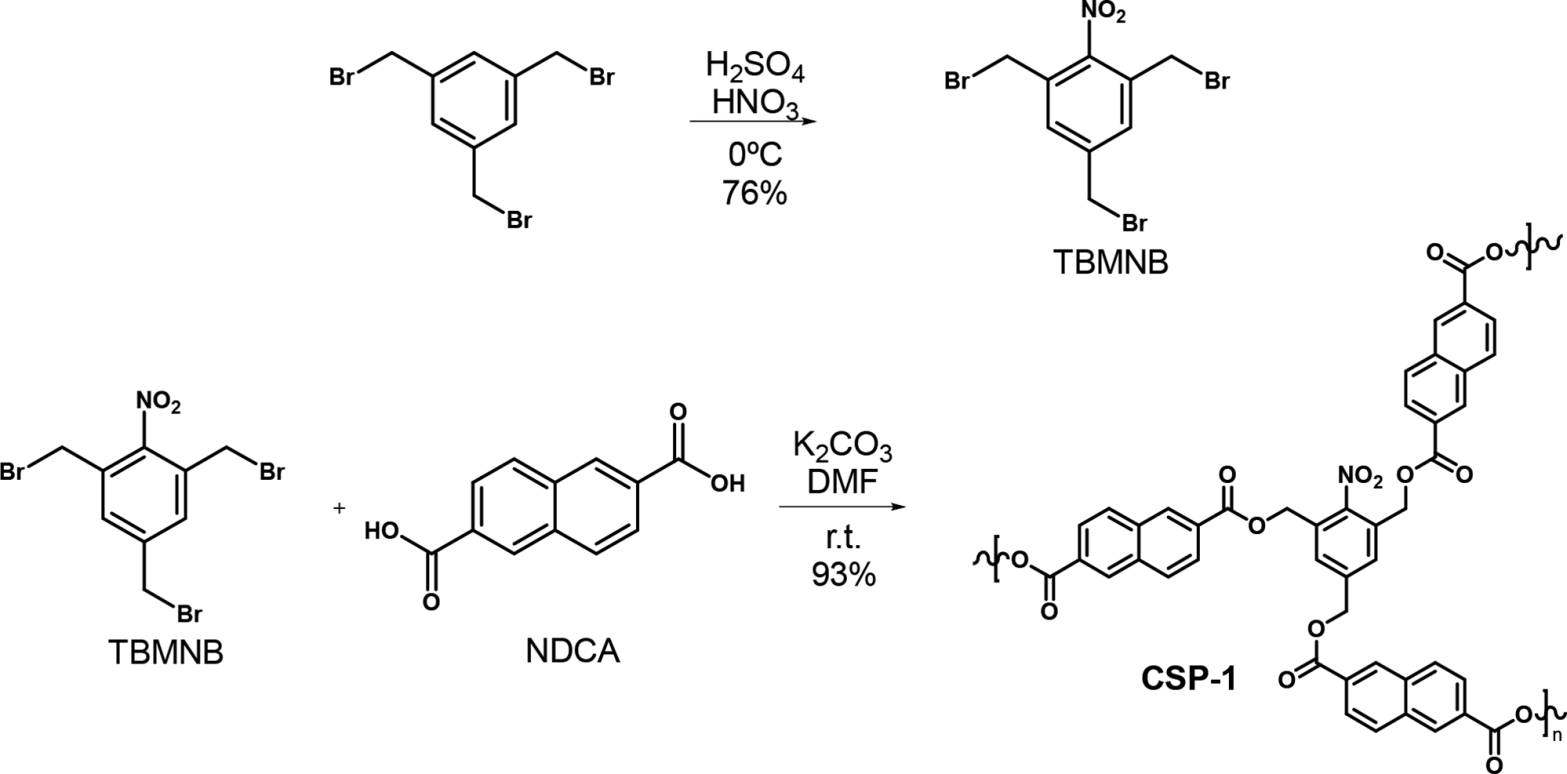
Synthesis of **CSP-1**

**CSP-1** was designed to include a self-immolative triple node, bearing a nitro group as a masked aniline. Additionally, an aromatic linker between the degradable monomers allows π–π interactions with the target analytes, which also have an aromatic ring. **CSP-1** was easily synthesized by a step growth polymerization from 1,3,5-tris(bromomethyl)-2-nitrobenzene (TBMNB) and 2,6-naphthalene dicarboxylic acid (NDCA) in an excellent 93% yield (Scheme 2). The fine white powder obtained was insoluble in several solvents tested: different amounts of the polymer were mixed with deuterated ACN, MeOH, acetone, dimethyl sulfoxide (DMSO) and water. The NMR spectra showed no signal.

**CSP-1** was characterized by Fourier transformed infrared spectroscopy (FTIR), thermogravimetric analysis (TGA), X-ray diffraction spectroscopy (XRD), scanning electron microscopy (SEM), and the Brunauer–Emmett–Teller (BET) method. Figure S1 of the Supporting Information shows the comparative FTIR spectra of NDCA, TBMNB, and **CSP-1**, confirming that the reaction is almost complete. After the synthesis of each batch of **CSP-1**, the FTIR spectra were obtained in order to control the final product obtained. As an example, Figure S2 of the Supporting Information shows a comparison of the spectra obtained for the CSP synthesized in different batches, showing an excellent matching between them. Powder XRD experiments confirmed that **CSP-1** is an amorphous material (Figure S3 of the Supporting Information). The thermal stability of the polymer was also studied by TGA up to 800 °C, which showed that the polymer was stable until 220 °C (Figure S4 of the Supporting Information). The same TGA curve was obtained from **CSP-1** synthesized in different batches. The surface area and porosity of the resulting polymer were calculated through the N_2_ adsorption isotherm, whose plot is shown in Figure S5 of the Supporting Information. As shown in the figure, the polymer possessed a type II nitrogen gas adsorption isotherm (*c* = 137), which corresponds to a monolayer–multilayer adsorption process. The first monolayer is completed after the adsorption of 4 cm^3^ of N_2_ per gram of sample at low relative pressure (*P*/*P*^0^ < 0.05), and then, a continuous increase is shown by the superposition of the successive layers. The BET surface area was 21.8 m^2^/g (4.1 m^2^/g as standard deviation) calculated as mean of five determinations. Regarding the pore volume, it was 0.0382 cm^3^/g (0.0084 cm^3^/g as standard deviation) with an average pore width of 70.0 Å (7.00 nm, 0.7 nm as standard deviation). According to the IUPAC definition,^25^ this is a mesoporous material since it has pore diameters between 2 and 50 nm. The polymer microscopic morphology and uniformity were also examined by SEM. Figure S6 of the Supporting Information includes the SEM image of a representative sample of **CSP-1** in which it can be seen that the morphology of the polymer is very irregular, with variable sizes of particles (less than 1.5 μm) as well as particle aggregation. **CSP-1** obtained in different batches showed identical morphologies.

### Potentiometric Acid–Base Titration and Water Solubility at Different pH Values

Since the polymerization reaction may have left carboxylic acids as terminal groups, the new polymer is indeed a weak polyelectrolyte. Due to the chain connectivity, neighboring ionizable groups are close to each other, and there are strong interactions between them. As a result, their pH-dependent ionization is much more complex than the ionizable behavior of small molecules like, for example, acetic acid.^26^ For instance, polyelectrolytes hardly reach 50% ionization when the pH is p*K*_a_+1 (a weak acid at such pH that it is practically fully ionized). Furthermore, the ionization of this macromolecule produces expansion of its conformation at the same time that the counterions or other small ions around the chain change their distributions;^26,27^ the polymer charge is strong enough to overcome the chain hydrophobicity. A widespread experimental approach to characterize polyacids is to perform titration experiments, i.e., potentiometric titration, which are among the simplest and most useful experimental tools for probing the degree of neutralization of a polymeric acid. By applying the procedure described in the Experimental Section and plotting the Gran plot (*V*_b_ × 10^–pH^ vs *V*_b_, where *V*_b_ is the base volume),^28^ it was found that the mean p*K*_a_ value was 4.91 ± 0.04 (standard deviation). The coefficients of determination (*R*^2^) were above 0.99. The sites with such p*K*_a_ values can be assigned to the carboxyl group of NDCA which has a p*K*_a_ value around 3.69.^29^

The possibility of the increase of the solubility of the polymer at high pH values as a result of the expansion of its conformation was also studied by dispersing 40 mg of the polymer in 5 mL of water at different pH values, from 2 to 14. As can be seen in Figure S7 of the Supporting Information, the polymer was completely dissolved above pH 13.80 at room temperature, when the polymer charge is strong enough to overcome chain hydrophobicity. Experiments below pH 13 always had the same results: no apparent dissolution of the polymer. As a result, the polymer is stable enough at a wide range of pH values to allow its use as an extraction sorbent from water samples of different pH values.

### Adsorption Isotherm

The adsorption capability of the synthesized polymer toward BBP, one of the model analytes selected, was studied applying the procedure described in the Experimental Section. Figure S8 of the Supporting Information shows the adsorption curve, while Table S3 of the Supporting Information shows the Langmuir and Freundlich parameters of the adsorption isotherms of BBP onto the synthesized **CSP-1**. As can be seen, the adsorption isotherm fits better to a Langmuir model, that is, the formation of an adsorption monolayer on the porous surface (no multilayers are formed).^30^ According to the adsorption curve, the amount of analyte adsorbed increases as its initial concentration increases, probably due to an increase in the interactions between the surface of the sorbent and the molecules of the target compound. As a consequence, the number of active centers in the sorbent occupied by the molecules of the analyte increases the concentration of the analyte, which implies a dense packing of the organic compounds on the surface and in the structure of the adsorbent.^30^ After the results were obtained, it was observed that the adsorption of BBP from water solutions is favorable.

### GC-MS Conditions

**Figure 2:**
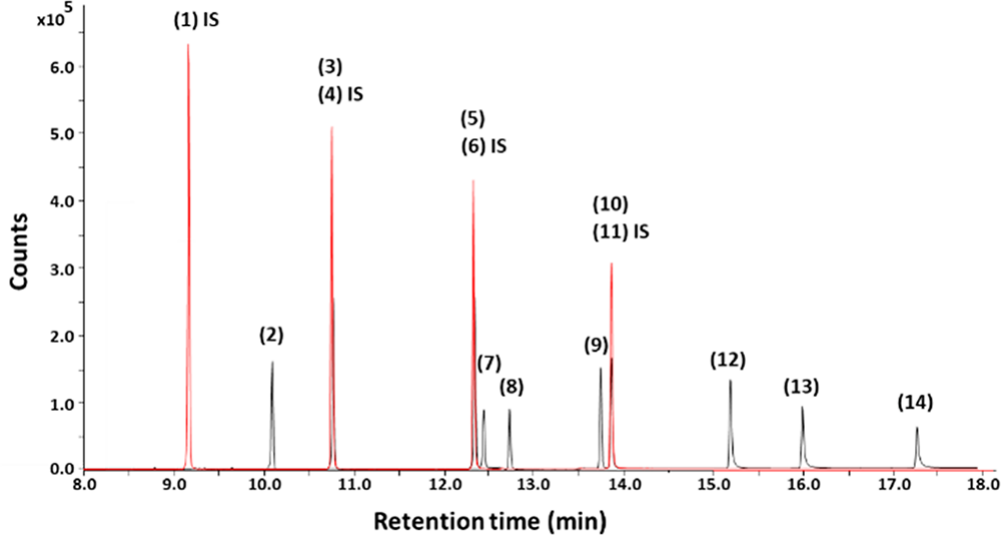
GC-MS chromatogram obtained under SIM mode of a working solution containing both analyte standards and the ISs dissolved in cyclohexane. Column: HP-5ms Ultra Inert ((5%-phenyl)-methylpolysiloxane, 30 m × 250 μm × 0.25 μm). Flow rate: 1.2 mL/min. Injection volume: 2 μL. Concentration of all the analytes and the ISs: 150 μg/L. Peak identification: DBP-*d*_4_ (1, IS), DIPP (2), DNPP (3), DNPP-*d*_4_ (4, IS), DHP (5), DHP-*d*_4_ (6, IS), BBP (7), DEHA (8), DCHP (9), DEHP (10), DEHP-*d*_4_ (11, IS), DNOP (12), DINP (13), and DIDP (14).

In this work, a group of nine PAEs and one adipate was selected as model analytes and analyzed by GC-MS, achieving an acceptable separation through the thermal gradient described in the Experimental Section. In this case, it is necessary to have all PAEs perfectly separated because all of them have the same quantifier ion (149 *m*/*z*),^31^ as can be seen in Table S2 of the Supporting Information. Four ISs widely distributed throughout the chromatogram were used: DBP-*d*_4_ for BBP; DNPP-*d*_4_ for DIPP and DNPP; DHP-*d*_4_ for DHP, DEHA, and DCHP; and DEHP-*d*_4_ for DEHP, DNOP, DINP, and DIDP. In Figure 2, a chromatogram of a working solution of the target analytes is shown, where it is also observed that the short-chain analytes elute first, and the long-chain ones elute last. Once the conditions of the GC-MS system were established, instrumental calibration was performed adding the ISs at a concentration of 125 μg/L. Table S4 of the Supporting Information shows the linear range studied for each analyte and the R^2^ values (higher than 0.9949 in all cases), as well as the slope and the intercept values with their respective confidence intervals. In addition, the instrumental limits of detection (LODs) and quantification (LOQs) are also shown for each studied compound, calculated considering three and 10 times the signal/noise ratio, respectively, which were experimentally verified. In order to test the repeatability of the injection and the separation, intraday and interday precision studies were developed by monitoring both peak areas and the retention times. For this purpose, standard solutions of the target analytes at three different concentration levels (10, 125, and 250 μg/L) were injected five times on the same day and on three different days (*n* = 15). Intraday relative standard deviation (RSD) values were below 4.5% and 0.04% for peak areas and retention times, respectively, while interday precision was below 11.9% for peak areas and lower than 0.03% for retention times.

### Optimization of the μdSPE Procedure

Once the polymer was synthesized, it was applied as a sorbent in a μdSPE procedure for the extraction of the selected target analytes from water samples. This extraction mode was selected, since it clearly simplifies the extraction procedure, making it faster and with a lower consumption of sorbents and solvents compared to classical SPE. For this purpose, and in order to obtain the best extraction conditions, sample pH, agitation type and time, sorbent amount, and solvent type and volume were initially optimized using 50 mL of Milli-Q water in order to avoid a matrix effect (ME) during optimization. The optimized parameters were evaluated through absolute recovery values. For this purpose, samples were spiked with the analytes and the ISs at concentrations of 125 μg/L before or after the extraction procedure.

### Sample pH Effect

To evaluate the effect of pH, 30 mg of the sorbent were weighed and put in contact with 50 mL of Milli-Q water at different pH values (between 3 and 8). After manual agitation for 2.5 min, the sample was passed through a glass column containing two PTFE frits at the bottom. Subsequently, the sorbent was packed by placing another PTFE frit on top and dried under vacuum for 25 min. Finally, the previously retained analytes were eluted with 15 mL of EtOAc (always maintaining a flow rate of 1 mL/min), and the solvent was evaporated to dryness (40 °C, 180 mbar) and reconstituted with 400 μL of cyclohexane. The extract obtained was filtered through a 0.22 μm PVDF disc filter before injection in the GC-MS system. Three extractions were made at each pH value. Figure S9 of the Supporting Information shows the variation of the absolute recovery values versus the different pH values, as well as the standard deviation of each of them. As can be seen, at pH above 4.0–5.0, the recovery values of some of the analytes generally decrease. Since the pH of the sample cannot have a great influence on the extraction of the target analytes as a result of the absence of any ionizable moiety, such a decrease might be caused by the negative charge of the polymer at pH values above its estimated p*K*_a_ value. Therefore, pH was adjusted to 4.0 in all the samples for further analyses to guarantee a neutral charge of the polymer, which at the same time provided the highest recovery values for most of the analytes.

### Agitation Type and Time Effects

In order to study the effects of the agitation type and time, the sorbent was dispersed in the aqueous sample by manual shaking or using ultrasound agitation for 1, 2.5, and 5 min, carrying out each extraction in triplicate. Manual shaking for 2.5 min clearly provided higher absolute recovery values (between 48% and 92%) than those obtained using ultrasounds or other extraction times, which were below 59%. Therefore, manual agitation was applied, which does not require additional energy consumption.

### Sorbent Amount Effect

The effect of the amount of polymer was also studied in triplicate considering 20, 30, 40, and 50 mg under the previously optimized extraction conditions: 50 mL of Milli-Q water at pH 4.0 and manual agitation for 2.5 min. Figure S10 of the Supporting Information shows the results obtained, in which it can be deduced that 30 mg were sufficient to achieve a quantitative extraction for the studied compounds, since the absolute recovery values were between 62% and 94% for all the analytes, except for BBP (the analyte with the lowest log *K*_OW_ value) which was of 50%.

### Elution Solvent Type and Volume Effect

Different organic solvents (i.e., MeOH, acetone, ACN, and EtOAc) were also tested for the elution of the target analytes. As shown in Figure S11 of the Supporting Information, MeOH hardly elutes the analytes (absolute recovery values were lower than 48%), while ACN and EtOAc provided better results with recovery values in the range of 48%–86%, except in the case of BBP, which had a recoveries of 34% and 38%, respectively. Since EtOAc provided slightly higher recovery values for some of the analytes, it was selected.

**Figure 3:**
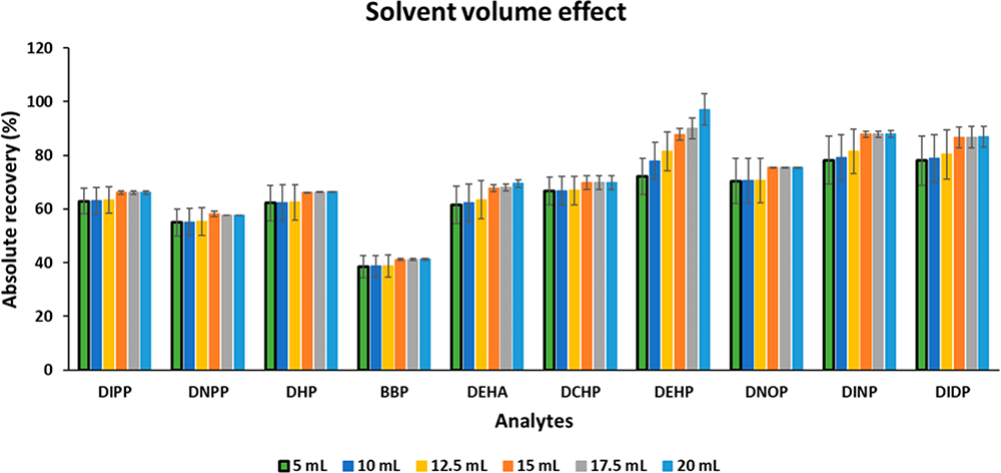
Effect of the solvent volume used in the elution step on the peak areas of the selected PAEs. Extraction conditions: 30 mg sorbent, 50 mL of spiked Milli-Q water at 125 μg/L (pH 4.0), manual shaking for 2.5 min, and elution with EtOAc.

Likewise, different volumes of EtOAc were tested (5, 10, 12.5, 15, 17.5, and 20 mL), finding that 5 mL was sufficient for a quantitative extraction of the target analytes and that the extraction at higher volumes did not show significant differences (Figure 3). In the case of DEHP, a slightly different pattern was observed (at higher volumes, the recovery increased), but after elution with 5 mL, its recovery value was high enough (72%) to allow the use of a much lower amount of solvent. It should also be indicated that μdSPE is frequently a nonexhaustive procedure. Therefore, very high absolute recovery values are not always obtained, even though in our case such values are above 60% for eight of the 10 analytes, which can be considered appropriate for a μdSPE procedure.

In order to test the interbatch reproducibility of the extraction, **CSP-1** synthesized in three different batches was applied to extract in quintuplicate (*n* = 5) Milli-Q water samples spiked with the target analytes at the same extraction levels. Similar recovery values (between 41% and 73%) with good RSD values (between 7% and 16%) were obtained for the three batches, showing (together with FTIR and SEM measurements) that the synthesis procedure is highly reproducible.

### Trueness Evaluation and Method Calibration

Once the μdSPE procedure was optimized, the method was applied to the extraction of the selected plasticizers from tap, waste, and spring water samples. For this purpose, matrix-matched calibration and recovery studies were carried out. Since PAEs are ubiquitous in any laboratory,^32^ procedural and sample blanks were also analyzed, and the concentration (if any) was subtracted from that found in the samples.

Samples spiked at three concentration levels (20, 75, and 150 μg/L for all the analytes and samples, and 125 μg/L for the ISs; concentration in the final extract) were studied to determine the trueness of the proposed methodology once applied to the analyses of the different types of water samples performing five consecutive replicates (*n* = 5) at each level. Table S5 of the Supporting Information shows the relative recovery values calculated by comparing the relative peak areas of the spiked samples with the relative peak areas of standards (spiked blank final extracts), which were between 70% and 120% for most of them, with satisfactory RSD values, below 18%. The LOQs of the method, which are also shown in the table, ranged between 6.77 and 139 ng/L. Such values were also experimentally verified.

On the other hand, a matrix-matched calibration was carried out due to the possible existence of a ME that may cause a suppression or an increase of the GC-MS signal. For this purpose, cyclohexane solutions of the target analytes and ISs at different concentrations were injected in triplicate. The ISs as well as the studied compounds were added after the extraction procedure, always keeping the ISs at 125 μg/L (concentration in the final extract). The linear range and the confidence intervals of the calibration curves, as well as the *R*^2^ values which were higher than 0.995, are shown in Table S6 of the Supporting Information. This table also shows the values of the ME calculated using the following equation^33^
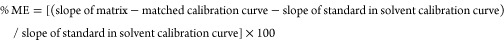


As can be seen, a high signal enhancement (>50%) was appreciated for some analytes, especially for DNOP, DINP, and DIDP, but also for DIPP, BBP, and DEHA. While others, such as DNPP and DHP, showed a moderate ME (20%–50%) in all the matrices studied. These results clearly indicate the need to take ME into account for further studies and, therefore, the need to perform matrix-matched calibration.

### Analysis of Different Water Samples

Finally, in order to demonstrate the applicability of the developed μdSPE procedure, four tap water samples, six waste water samples, and two spring water samples were analyzed. pH and conductivity values of the samples were measured and compiled in Table S7 of the Supporting Information for comparison purposes. Samples were analyzed without further treatment, except for the waste water samples which were previously filtered through a PVDF filter with a pore size of 0.22 μm. The results are compiled in Table 1, where it is observed that DBP and DEHP were detected and quantified in some of the spring and waste water samples, while BBP was found in one waste water sample but below the LOQ of the method.

**Table 1:** Results of Analysis of Different Water Samples after **CSP-1**–μdSPE–GC-MS Procedure

		Analytes (μg/L)		
Matrix	Sample	DBP	BBP	DEHP
Tap water	1	n.d.	n.d.	n.d.
	2	n.d.	n.d.	n.d.
	3	n.d.	n.d.	n.d.
	4	n.d.	n.d.	n.d.
				
Waste water	1	n.d.	n.d.	n.d.
	2	n.d.	n.d.	n.d.
	3	n.d.	n.d.	n.d.
	4	n.d.	n.d.	0.15 ± 0.04
	5	0.22 ± 0.03	<LOQ	0.68 ± 0.04
	6	n.d.	n.d.	n.d.
				
Spring water	1	n.d.	n.d.	n.d.
	2	0.17 ± 0.04	n.d.	n.d.

Table S8 of the Supporting Information shows a comparison between the present work and those already published in the literature in which the extraction of plasticizers from different water samples has been carried out by dSPE using different sorbents. As an example, several plasticizers were extracted from mineral water samples packaged in plastic bottles, using metal–organic frameworks (MOF-70 and TMU-6)^34,35^ and dummy molecularly imprinted microbeads as sorbents.^36^ Graphene has also been used as a sorbent for the extraction of PAEs from drinking and environmental water samples.^37,38^ These works used GC for the separation of the target compounds coupled to a flame ionization detector and a mass spectrometer showing higher LODs than those obtained in the present work (0.002–0.042 μg/L). Cheng et al.^39^ determined DEHP in river, lake, and rain waters using 20 mg of a molecular imprinted polymer in combination with graphene oxide for their dSPE before GC-MS determination. Although the matrices were different from those studied in the present work, recovery values were similar to those obtained in this work for that analyte but with higher LODs.

On the other hand, other works in which LC coupled to an MS detector have also been applied for the extraction of several PAEs from water samples^40,41^ have recovery percentages comparable to those of our work but with a slightly higher sensitivity. As an example, González-Sálamo et al.^40^ used 120 mg of a commercial MOF (Basolite F300) as a sorbent for the extraction of eight PAEs and the adipate DEHA, finding a concentration of DEHP in waste water similar to that found in one of the waste water samples of our study, which were collected from the same point. In this case the LODs were in the 6.6–20.7 ng/L range. Vivas et al.^41^ developed a μdSPE–LC-MS procedure for the determination of 14 endocrine-disrupting chemicals (including six PAEs) from bottled mineral water. In this case, the authors used 80 mg of C_18_ as the sorbent (LODs between 1.6 and 23.2 ng/L).

### Polymer Degradation and Recycling

**Scheme 3:**
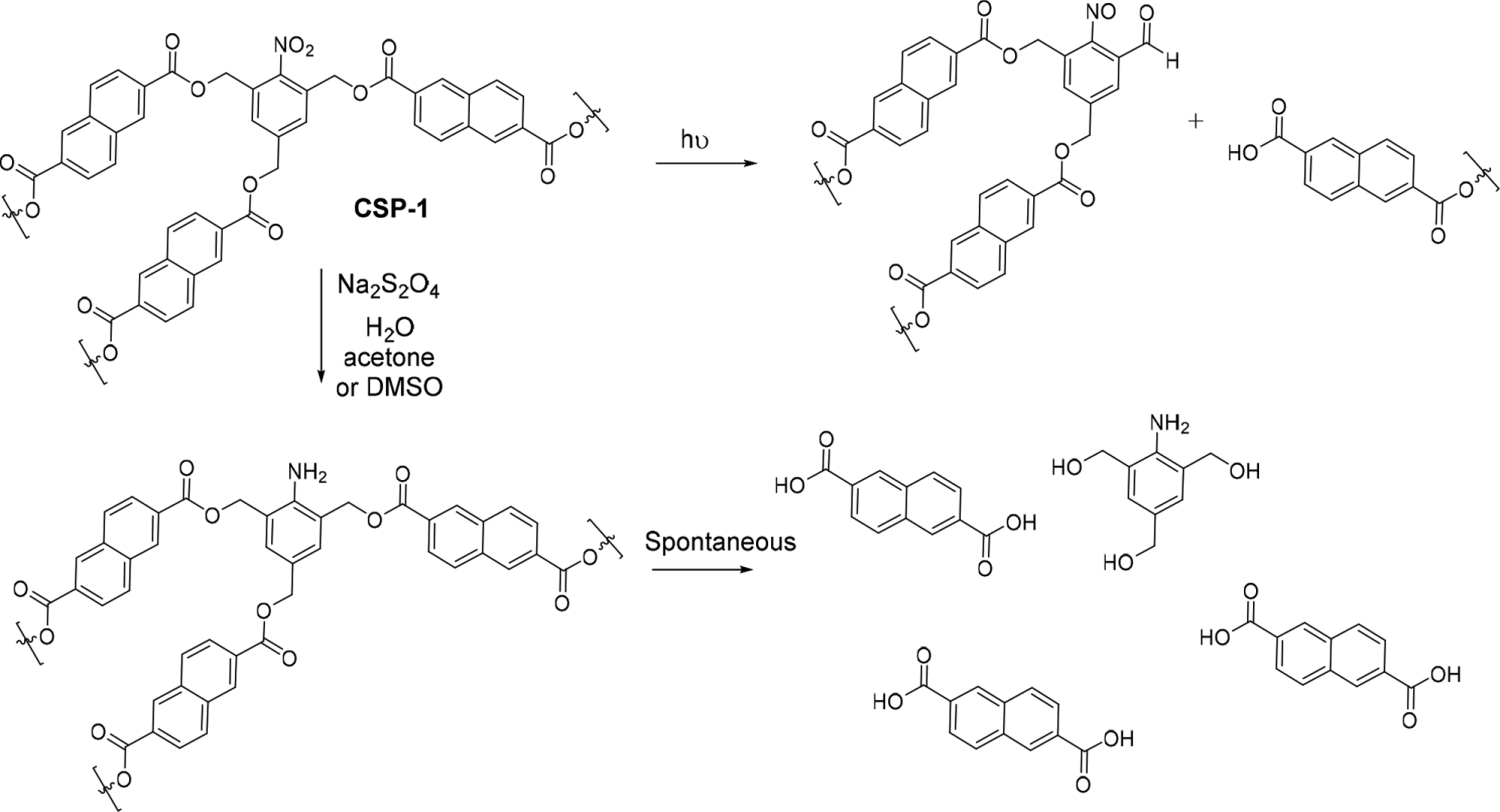
Photodegradation and Self-Immolation Mechanisms of **CSP-1**

The self-immolative units of **CSP-1** display a nitro group as a masked aniline, and therefore, they are designed to be disassembled in reductive media. Curiously, *ortho*-nitrobenzyl is a well-known photoactivatable protecting group that can be broken by UV irradiation.^42^ It was precisely the photocleavage of the first degradation method to be tested. A suspension of **CSP-1** in DMSO-*d*_*6*_ was irradiated with an ACE-Hanovia photochemical lamp 7830–60 (450W) and monitored by ^1^H NMR. After 1.5 h, the solid had disappeared, and the solution turned yellow. The ^1^H NMR of the solution showed peaks characteristic of the naphthalene linker and other degradation products, although peaks were not sharp or defined enough. Indeed, photocleavage of the polymer only breaks one of the *ortho* bonds of the self-immolative unit, and therefore, different soluble oligomers were probably obtained (Scheme 3).

More interestingly, when a suspension of **CSP-1** was reduced with sodium dithionite, again the solid disappeared, and a clear solution was obtained (Scheme 3). In this case, the ^1^H NMR showed the sharp peaks of the naphthalene linker, which implies a much more efficient degradation than the previous one. Importantly, the recovery of the naphthalene monomer could be measured, proving that these kinds of polymers are not only efficient sorbents but also easily recyclable materials. Detailed information can be found in the Supporting Information.

### Degradation–Depolymerization Cycle Study

In order to check the possible repolimerization of the polymer, a reductive degradation with sodium dithionite (335 mg) starting from 100 mg of polymer **CSP-1** in 20 mL of acetone:water (9:1, v/v) was carried out. After 12 h of reflux, the polymer was degraded. The reaction was cooled to room temperature. The solvents were evaporated, and the solid was vacuum-dried. A portion of such a crude residue was dissolved in DMF, and 27 mg of TBMNB and 32 mg of K_2_CO_3_ were added to the reaction flask. The reaction was performed as explained before. A white polymer was obtained, which gives an IR spectrum that matched that of the original polymer (Supporting Information), confirming that the crude mixture obtained after degradation can be used for repolymerization of **CSP-1**.

## Conclusions

In this work, a chain-shattering polymer (**CSP-1**), easily degradable by the right stimulus, has been successfully applied as a recyclable μdSPE sorbent. **CSP-1** is mesoporous and stable in nearly all water pH ranges. Up to 220 °C, it is quite insoluble in many common solvents, and it is able to establish noncovalent aromatic interactions, all of which support its use as a μdSPE sorbent. In fact, good recovery values with acceptable RSD values were obtained for a group of plasticizers selected as model analytes and extracted from different water samples (tap, waste, and spring waters) after the optimization of the extraction procedure. Concerning the polymer degradability, **CSP-1** was easily photocleaved by UV irradiation into soluble oligomers. More interestingly, reduction with sodium dithionite led to a complete degradation of the polymer and the recovery of the constituent monomers.

This proof-of-concept work suggests that CSPs, if properly designed, could be used as extraction sorbents with an evident value from a sustainability point of view. Since they are modular, they could be designed to be degraded by different kinds of stimuli, from pH variation to UV radiation, enzymatic digestion, and more, allowing their recycling. Furthermore, they have a huge potential of variability both in structure and in degradation pathways, which is also translated into affinity for different families of chemical compounds and different ways of recycling, respectively. Besides, degradation kinetics could also be modulated, providing faster degradation rates than other degradable polymers.

Further studies with natural, nontoxic monomers will get these sorbents much closer to the optimal environmentally friendly procedures that the analytical chemistry field is currently aiming for.

## Supporting Information Available

Calibration graphs, results of the recovery study, synthesis procedure, results of the characterization of the polymeric material, and more (PDF)
