# Supplementary material for: Chain-Shattering Polymers as Degradable Microdispersive Solid-Phase Extraction Sorbents
Source: Anal Chem. 2022 Jun 13;94(25):9065–73. doi: 10.1021/acs.analchem.2c01301 (PMC9244869; doi:10.1021/acs.analchem.2c01301)
Supplement: Supplementary file 1 — ac2c01301_si_001.pdf [file ac2c01301_si_001.pdf]

SUPPORTING INFORMATION

Chain-shattering polymers as degradable microdispersive solid-phase extraction sorbents

Cecilia Ortega-Zamora[1,2]*, Javier González-Sálamo*[1,2,3]*, Marcelle D. Perretti*[4]*,*

David Santana[4]*, Romen Carrillo*[4,**] *and Javier Hernández-Borges*[1,2*]

[1]*Departamento de Química, Unidad Departamental de Química Analítica, Facultad de Ciencias, Universidad de La Laguna (ULL). Avda. Astrofísico Fco. Sánchez, s/n. 38206 San Cristóbal de La Laguna, Spain.*

[2]*Instituto Universitario de Enfermedades Tropicales y Salud Pública de Canarias, Universidad de La Laguna (ULL). Avda. Astrofísico Fco. Sánchez, s/n. 38206 San Cristóbal de La Laguna, Spain.*

[3]*Department of Chemistry, Sapienza University, P.le Aldo Moro, 5. 00185 Rome, Italy.*

[4]*Instituto de Productos Naturales y Agrobiología, CSIC. Avda. Astrofísico Fco. Sánchez, s/n. 38206 San Cristóbal de La Laguna, Spain.*

email: jhborges@ull.edu.es*

*email**: rcarrillo@ipna.csic.es*

TABLE OF CONTENT

Table S1.- Chemical structure and properties of the studied PAEs and DEHA (pages S3-S4).

Table S2.- GC-MS retention times, quantifier and qualifier m/z values of DEHA and the selected PAEs and ISs (page S5).

Table S3.- Langmuir and Freundlich parameters of the adsorption isotherms of BBP onto the synthesized polymer (page S6).

Table S4.- Internal instrumental calibration data of the target analytes (page S7).

Table S5.- Relative recovery and RSD values of the target analytes in tap, waste and spring water (pages S8-S9).

Table S6.- Matrix-matched calibration data of the target analytes in tap, waste and spring water (pages S10-S11).

Table S7.- pH and conductivity data of the water samples analyzed in this work (page S12).

Table S8.- Previous studies in which a dSPE procedure has been applied for the extraction of plasticizers from water samples (pages S13-S15).

Polymer synthesis (pages S16-S17).

Degradation studies and RMN spectra (pages S18-S26).

Degradation-repolymerization cycle study (S27).

Figure S1.- FTIR spectra of NDCA, TBMNB and the CSP-1 (page S28).

Figure S2.- FTIR spectra of the CSP-1 synthesized in different batches (page S29).

Figure S3.- X-ray diffraction pattern obtained for the polymer showing the shape of a predominantly amorphous material (page S30).

Figure S4.- TGA curve of the CSP-1 (page S31).

Figure S5.- N_2 adsorption isotherm plot of the CSP-1 (page S32).

Figure S6.- SEM images of the CSP-1 (page S33).

Figure S7.- Photograph of the resulting mixtures of 40 mg of the polymer and 5 mL of water at pH 11, 12, 13 and 14 (page S34).

Figure S8.- BBP HPLC-UV calibration curve (top) and adsorption of BBP onto CSP-1 (down) (page S35).

Figure S9.- Effect of the pH during the extraction step on the peak areas of the target PAEs. Extraction conditions: 30 mg sorbent, 50 mL of spiked Milli-Q water at 125 μg/L, manual agitated for 2.5 min and elution with 15 mL of ethyl acetate (page S36).

Figure S10.- Effect of the sorbent amount used in the extraction step on the peak areas of the selected PAEs. Extraction conditions: 50 mL of spiked Milli-Q water at 125 μg/L (pH 4.0), manual agitated for 2.5 min, and elution with 15 mL of ethyl acetate (page S37).

Figure S11.- Effect of the solvent used in the elution step on the peak areas of the selected analytes. Extraction conditions: 30 mg sorbent, 50 mL of spiked Milli-Q water at 125 μg/L (pH 6.0), manual agitated for 2.5 min, and elution with 15 mL of each solvent (page S38).

Table S1.- Chemical structure and properties of the studied PAEs and DEHA.

**Analyte**	**Structure**	**Molecular formula**	**MM (g/mol)**	**Solubility in water (g/L, 25 ºC)**	**Vapor pressure (mmHg, 25 ºC)**	**Log K_{OW}**	**Melting point (ºC)**	**Boiling point (ºC)**
DIPP	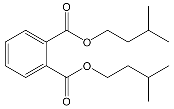	$C_{18}H_{26}O_4$	306.2	0.20	3.54·10-4[a]	5.50[b]	-	336
DNPP	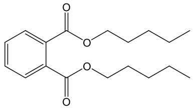	$C_{18}H_{26}O_4$	306.2	0.0008	$2.8·10^{-5}$[b]	5.62	-55	342
DHP	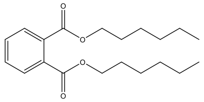	$C_{20}H_{30}O_4$	334.4	0.00005	$1.40·10^{-5}$	6.82	-58	302
BBP	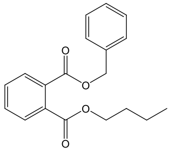	$C_{19}H_{20}O_4$	312.1	2.69	$8.25·10^{-6}$	4.73	-35	370
DEHA	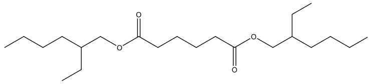	$C_{22}H_{42}O_4$	370.6	0.00078[a]	$8.5·10^{-7}$	6.11	-68	335
DCHP	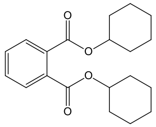	$C_{20}H_{26}O_4$	330.2	4.0[c]	$8.69·10^{-7}$	6.20	66	225
DEHP	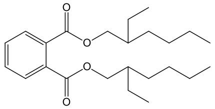	$C_{24}H_{38}O_4$	390.3	0.00027	$1.42·10^{-7}$	7.60	-55	230

Table S1.- (Continued).

Analyte	Structure	Molecular formula	MM (g/mol)	Solubility in water (g/L, 25 ºC)	Vapor pressure (mmHg, 25 ºC)	Log K_{OW}	Melting point (ºC)	Boiling point (ºC)
DNOP	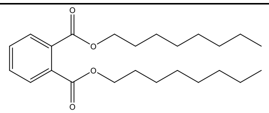	$C_{24}H_{38}O_4$	390.6	0.000022	$1.0·10^{-7}$	8.20	-25	385
DINP	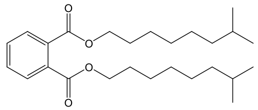	$C_{26}H_{42}O_4$	419.3	0.0002[d]	$5.40·10^{-7}$	9.37	-48	406
DIDP	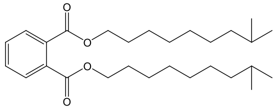	$C_{28}H_{46}O_4$	446.3	0.00028	$5.28·10^{-7}$	10.36	-50	423

[a] 22 ºC. [b] Predicted value. [c] 24 ºC. [d] 20 ºC. Data taken from SciFinder® and PubChem databases. MM: Molecular mass.

Table S2.- GC-MS retention times, quantifier and qualifier m/z values of DEHA and the selected PAEs and ISs.

Analyte	Retention time (min)	RSD values (%, n=60 injections)	Quantifier (m/z)	Qualifier 1 (m/z)	Qualifier 2 (m/z)
DBP-d_4	9.16	0.04	153	209	227
DIPP	10.08	0.04	149	237	219
DNPP-d_4	10.75	0.04	153	223	241
DNPP	10.75	0.04	149	219	237
DHP-d_4	12.33	0.04	153	255	237
DHP	12.33	0.03	149	251	233
BBP	12.43	0.04	149	91	206
DEHA	12.72	0.03	129	112	147
DCHP	13.79	0.03	149	167	249
DEHP-d_4	13.91	0.03	153	283	171
DEHP	13.91	0.03	149	167	279
DNOP	15.28	0.03	149	167	279
DINP	16.11	0.03	149	167	293
DIDP	17.44	0.03	149	167	307

Ionization energy of -70 eV in all cases.

Table S3.- Langmuir and Freundlich parameters of the adsorption isotherms of BBP onto the synthesized polymer.

**Langmuir**		
**q_{max} (mg/g)**	**K_L (L/mg)**	**R^2**
579.86	0.17	0.9996
**Freundlich**		
**K_F (L/m^2)**	**1/n**	**R^2**
156.46	0.30	0.9243

Table S4.- Internal instrumental calibration data of the target analytes (DBP-d_4 was used as IS of BBP, DNPP-d_4 was used as IS of DIPP and DNPP, DHP-d_4 was used as IS of DHP, DEHA and DCHP, while DEHP-d_4 was used as IS of DEHP, DNOP, DINP and DIDP).

Analytes	Studied linear range (µg/L)	Regression equation (n=8)		$s_{y/x}$	R^2	LOD (µg/L)	LOQ (µg/L)
		$b \pm s_b \cdot t_{(0.05;6)}$	$a \pm s_a \cdot t_{(0.05;6)}$				
DIPP	0.5-150	$5.20 \cdot 10^{-3} \pm 2.40 \cdot 10^{-4}$	$-1.34 \cdot 10^{-2} \pm 1.79 \cdot 10^{-2}$	0.0156	0.9979	0.2	0.5
DNPP	0.5-150	$9.82 \cdot 10^{-3} \pm 2.76 \cdot 10^{-4}$	$-1.61 \cdot 10^{-2} \pm 2.06 \cdot 10^{-2}$	0.0180	0.9992	0.2	0.5
DHP	5-150	$1.14 \cdot 10^{-2} \pm 4.92 \cdot 10^{-4}$	$-3.88 \cdot 10^{-2} \pm 4.24 \cdot 10^{-2}$	0.0245	0.9990	0.4	1.2
BBP	5-150	$2.01 \cdot 10^{-3} \pm 2.00 \cdot 10^{-4}$	$-1.39 \cdot 10^{-2} \pm 1.72 \cdot 10^{-2}$	0.0100	0.9949	1.3	4.3
DEHA	5-150	$2.93 \cdot 10^{-3} \pm 2.50 \cdot 10^{-4}$	$-1.80 \cdot 10^{-2} \pm 2.15 \cdot 10^{-2}$	0.0124	0.9962	0.7	2.5
DCHP	5-150	$6.15 \cdot 10^{-3} \pm 3.61 \cdot 10^{-4}$	$-3.02 \cdot 10^{-2} \pm 3.11 \cdot 10^{-2}$	0.0180	0.9982	0.6	1.9
DEHP	5-150	$9.46 \cdot 10^{-3} \pm 2.88 \cdot 10^{-4}$	$-1.55 \cdot 10^{-2} \pm 2.48 \cdot 10^{-2}$	0.0143	0.9995	0.2	0.7
DNOP	5-150	$5.75 \cdot 10^{-3} \pm 3.16 \cdot 10^{-4}$	$-5.72 \cdot 10^{-4} \pm 2.72 \cdot 10^{-2}$	0.0157	0.9984	1.3	4.4
DINP	5-150	$3.88 \cdot 10^{-3} \pm 2.78 \cdot 10^{-4}$	$1.33 \cdot 10^{-3} \pm 2.40 \cdot 10^{-2}$	0.0139	0.9973	0.8	2.8
DIDP	5-150	$2.82 \cdot 10^{-3} \pm 2.77 \cdot 10^{-4}$	$4.60 \cdot 10^{-3} \pm 2.38 \cdot 10^{-2}$	0.0138	0.9950	0.6	2.0

Table S5.- Relative recovery and RSD (between parenthesis) values of the target analytes in tap, waste and spring water (n=5 at each spiking level).

Analytes	Sample	Level 1[a]	Level 2[a]	Level 3[a]	Mean recovery value % (RSD)	LOQ_{method} (ng/L)
		Recovery % (RSD %)	Recovery % (RSD %)	Recovery % (RSD %)		
DIPP	Tap water	116 (9)	113 (4)	107 (2)	112 (6)	6.77
	Wastewater	101 (4)	114 (6)	105 (6)	107 (7)	10.2
	Spring water	120 (6)	113 (8)	116 (6)	116 (7)	9.25
DNPP	Tap water	99 (9)	101 (1)	97 (1)	99 (5)	7.77
	Wastewater	94 (4)	99 (8)	107 (3)	100 (7)	11.0
	Spring water	109 (7)	95 (2)	100 (11)	101 (9)	10.6
DHP	Tap water	97 (3)	100 (2)	97 (2)	98 (2)	15.6
	Wastewater	95 (3)	106 (5)	106 (3)	102 (6)	20.4
	Spring water	116 (7)	97 (6)	108 (7)	107 (10)	21.1
BBP	Tap water	115 (6)	109 (7)	106 (5)	110 (7)	80.1
	Wastewater	113 (9)	116 (11)	125 (7)	118 (10)	139
	Spring water	119 (4)	83 (6)	120 (3)	107 (17)	119
DEHA	Tap water	98 (9)	120 (7)	106 (6)	108 (11)	29.1
	Wastewater	119 (6)	114 (6)	119 (2)	117 (5)	36.0
	Spring water	127 (6)	104 (3)	114 (10)	115 (11)	40.4
DCHP	Tap water	92 (8)	99 (7)	88 (4)	93 (8)	25.2
	Wastewater	75 (10)	85 (11)	73 (16)	78 (14)	41.5
	Spring water	102 (8)	96 (9)	111 (7)	103 (10)	34.2
DEHP	Tap water	77 (15)	63 (15)	99 (4)	80 (22)	8.30
	Wastewater	113 (9)	116 (8)	124 (8)	118 (9)	7.26
	Spring water	101 (12)	132 (8)	79 (6)	102 (21)	8.68

Table S5.- (Continued).

Analytes	Sample	Level 1[a] Recovery % (RSD %)	Level 2[a] Recovery % (RSD %)	Level 3[a] Recovery % (RSD %)	Mean recovery value % (RSD)	LOQ_{method} (ng/L)
DNOP	Tap water	101 (11)	100 (4)	93 (6)	98 (8)	45.0
	Wastewater	93 (6)	91 (8)	104 (4)	96 (9)	59.3
	Spring water	106 (5)	74 (5)	73 (8)	85 (20)	68.8
DINP	Tap water	118 (6)	105 (2)	99 (3)	107 (9)	26.6
	Wastewater	109 (3)	112 (7)	114 (6)	112 (6)	33.1
	Spring water	119 (5)	103 (5)	95 (4)	106 (11)	35.9
DIDP	Tap water	121 (2)	118 (4)	106 (6)	115 (7)	17.4
	Wastewater	105 (3)	109 (8)	119 (7)	111 (8)	23.3
	Spring water	114 (11)	98 (8)	98 (4)	103 (11)	25.7

[a]Level 1 of 0.16 µg/L, level 2 of 0.6 µg/L and level 3 of 1.2 µg/L for all the analytes in the samples.

Table S6.- Matrix-matched calibration data of the target analytes in tap, waste and spring water (DBP-d_4 was used as IS of BBP, DNPP-d_4 was used as IS of DIBP, DIPP and DNPP, DHP-d_4 was used as IS of DHP, DEHA and DCHP, while DEHP-d_4 was used as IS of DEHP, DNOP, DINP and DIDP).

Analytes	Sample	Studied linear range (µg/L)	Regression equation (n=8)		$s_{y/x}$	R^2	ME (%)*
			$b \pm s_b \cdot t_{(0.05;6)}$	$a \pm s_a \cdot t_{(0.05;6)}$			
DIPP	Tap water	0.5-150	$8.37 \cdot 10^{-3} \pm 3.56 \cdot 10^{-4}$	$-8.50 \cdot 10^{-3} \pm 2.65 \cdot 10^{-2}$	$2.32 \cdot 10^{-2}$	0.9982	61
	Wastewater	0.5-150	$8.40 \cdot 10^{-3} \pm 2.02 \cdot 10^{-4}$	$-1.09 \cdot 10^{-2} \pm 1.50 \cdot 10^{-2}$	$1.31 \cdot 10^{-2}$	0.9994	62
	Spring water	0.5-150	$8.15 \cdot 10^{-3} \pm 3.49 \cdot 10^{-4}$	$-1.72 \cdot 10^{-2} \pm 2.60 \cdot 10^{-2}$	$2.27 \cdot 10^{-2}$	0.9982	57
DNPP	Tap water	0.5-150	$1.25 \cdot 10^{-2} \pm 3.77 \cdot 10^{-4}$	$-2.03 \cdot 10^{-2} \pm 2.81 \cdot 10^{-2}$	$2.45 \cdot 10^{-2}$	0.9991	27
	Waste-wate	0.5-150	$1.27 \cdot 10^{-2} \pm 3.47 \cdot 10^{-4}$	$-2.15 \cdot 10^{-2} \pm 2.59 \cdot 10^{-2}$	$2.26 \cdot 10^{-2}$	0.9993	30
	Spring water	0.5-150	$1.22 \cdot 10^{-2} \pm 5.90 \cdot 10^{-4}$	$-2.54 \cdot 10^{-2} \pm 4.40 \cdot 10^{-2}$	$3.84 \cdot 10^{-2}$	0.9977	25
DHP	Tap water	5-150	$1.39 \cdot 10^{-2} \pm 3.86 \cdot 10^{-4}$	$-3.35 \cdot 10^{-2} \pm 3.32 \cdot 10^{-2}$	$1.92 \cdot 10^{-2}$	0.9996	22
	Wastewater	5-150	$1.43 \cdot 10^{-2} \pm 4.27 \cdot 10^{-4}$	$-4.11 \cdot 10^{-2} \pm 3.68 \cdot 10^{-2}$	$2.13 \cdot 10^{-2}$	0.9995	25
	Spring water	5-150	$1.38 \cdot 10^{-2} \pm 5.75 \cdot 10^{-4}$	$-2.72 \cdot 10^{-2} \pm 4.95 \cdot 10^{-2}$	$2.87 \cdot 10^{-2}$	0.9991	21
BBP	Tap water	5-150	$3.80 \cdot 10^{-3} \pm 2.39 \cdot 10^{-4}$	$-1.15 \cdot 10^{-2} \pm 2.06 \cdot 10^{-2}$	$1.19 \cdot 10^{-2}$	0.9979	89
	Wastewater	5-150	$4.29 \cdot 10^{-3} \pm 1.47 \cdot 10^{-4}$	$-1.98 \cdot 10^{-2} \pm 1.26 \cdot 10^{-2}$	$7.31 \cdot 10^{-3}$	0.9994	114
	Spring water	5-150	$3.95 \cdot 10^{-3} \pm 1.87 \cdot 10^{-4}$	$-1.47 \cdot 10^{-2} \pm 1.61 \cdot 10^{-2}$	$9.30 \cdot 10^{-3}$	0.9988	97
DEHA	Tap water	5-150	$4.90 \cdot 10^{-3} \pm 2.59 \cdot 10^{-4}$	$-1.23 \cdot 10^{-2} \pm 2.23 \cdot 10^{-2}$	$1.29 \cdot 10^{-2}$	0.9986	68
	Wastewater	5-150	$5.19 \cdot 10^{-3} \pm 3.18 \cdot 10^{-4}$	$-2.00 \cdot 10^{-2} \pm 2.74 \cdot 10^{-2}$	$1.59 \cdot 10^{-2}$	0.9981	77
	Spring water	5-150	$4.75 \cdot 10^{-3} \pm 2.05 \cdot 10^{-4}$	$-1.56 \cdot 10^{-2} \pm 1.77 \cdot 10^{-2}$	$1.02 \cdot 10^{-2}$	0.9990	62
DCHP	Tap water	5-150	$8.61 \cdot 10^{-3} \pm 4.02 \cdot 10^{-4}$	$-3.34 \cdot 10^{-2} \pm 3.46 \cdot 10^{-2}$	$2.00 \cdot 10^{-2}$	0.9989	40
	Wast-water	5-150	$7.21 \cdot 10^{-3} \pm 2.88 \cdot 10^{-4}$	$-2.22 \cdot 10^{-2} \pm 2.48 \cdot 10^{-2}$	$1.43 \cdot 10^{-2}$	0.9992	17
	Spring water	5-150	$7.61 \cdot 10^{-3} \pm 4.65 \cdot 10^{-4}$	$-1.87 \cdot 10^{-2} \pm 4.01 \cdot 10^{-2}$	$2.32 \cdot 10^{-2}$	0.9981	24
DEHP	Tap water	5-150	$1.24 \cdot 10^{-2} \pm 7.54 \cdot 10^{-4}$	$4.63 \cdot 10^{-1} \pm 6.49 \cdot 10^{-2}$	$3.76 \cdot 10^{-2}$	0.9981	31
	Wastewater	5-150	$1.54 \cdot 10^{-2} \pm 8.43 \cdot 10^{-4}$	$4.29 \cdot 10^{-1} \pm 7.26 \cdot 10^{-2}$	$4.20 \cdot 10^{-2}$	0.9985	63
	Spring water	5-150	$1.13 \cdot 10^{-2} \pm 7.69 \cdot 10^{-4}$	$5.58 \cdot 10^{-1} \pm 6.62 \cdot 10^{-2}$	$3.83 \cdot 10^{-2}$	0.9976	19
DNOP	Tap water	5-150	$1.84 \cdot 10^{-2} \pm 1.37 \cdot 10^{-3}$	$-1.08 \cdot 10^{-1} \pm 1.18 \cdot 10^{-1}$	$6.84 \cdot 10^{-2}$	0.9971	219
	Wastewater	5-150	$1.92 \cdot 10^{-2} \pm 9.71 \cdot 10^{-4}$	$-9.91 \cdot 10^{-2} \pm 8.36 \cdot 10^{-2}$	$4.84 \cdot 10^{-2}$	0.9987	234
	Spring water	5-150	$1.82 \cdot 10^{-2} \pm 1.37 \cdot 10^{-3}$	$-1.02 \cdot 10^{-1} \pm 1.18 \cdot 10^{-1}$	$6.81 \cdot 10^{-2}$	0.9971	217

Table S6.- Continued.

Analytes	Sample	Studied linear range (µg/L)	Regression equation (n=8)		$s_{y/x}$	R^2	ME (%)*
			$b \pm s_b \cdot t_{(0.05;6)}$	$a \pm s_a \cdot t_{(0.05;6)}$			
DINP	Tap water	5-150	$1.72 \cdot 10^{-2} \pm 6.58 \cdot 10^{-4}$	$1.92 \cdot 10^{-2} \pm 5.67 \cdot 10^{-2}$	$3.28 \cdot 10^{-2}$	0.9992	343
	Wast-water	5-150	$1.91 \cdot 10^{-2} \pm 4.34 \cdot 10^{-4}$	$-9.25 \cdot 10^{-3} \pm 3.73 \cdot 10^{-2}$	$2.16 \cdot 10^{-2}$	0.9997	394
	Spring water	5-150	$1.58 \cdot 10^{-2} \pm 1.43 \cdot 10^{-3}$	$5.25 \cdot 10^{-3} \pm 1.23 \cdot 10^{-1}$	$7.14 \cdot 10^{-2}$	0.9957	307
DIDP	Tap water	5-150	$1.37 \cdot 10^{-2} \pm 7.80 \cdot 10^{-4}$	$-5.86 \cdot 10^{-2} \pm 6.72 \cdot 10^{-2}$	$3.89 \cdot 10^{-2}$	0.9983	387
	Wastewater	5-150	$1.62 \cdot 10^{-2} \pm 8.65 \cdot 10^{-4}$	$-8.63 \cdot 10^{-2} \pm 7.45 \cdot 10^{-2}$	$4.31 \cdot 10^{-2}$	0.9985	473
	Spring water	5-150	$1.31 \cdot 10^{-2} \pm 6.72 \cdot 10^{-4}$	$-5.65 \cdot 10^{-2} \pm 5.79 \cdot 10^{-2}$	$3.35 \cdot 10^{-2}$	0.9986	363

Table S7.- pH and conductivity data of the water samples analyzed in this work.

	**pH**	**Conductivity (µS/cm) at 25 ºC**
	**Tap water**	
Sample 1	9.07	999
Sample 2	8.65	1030
Sample 3	9.03	595
Sample 4	7.77	641
	**Wastewater**	
Sample 1	8.69	1723
Sample 2	8.54	1360
Sample 3	7.85	1159
Sample 4	7.91	556
Sample 5	8.38	1440
Sample 6	8.51	1898
	**Spring water**	
Sample 1	9.34	1775
Sample 2	8.53	801

Sample 1 was also used for validation purpose.

Table S8.- Previous studies in which a dSPE procedure has been applied for the extraction of plasticizers from water samples.

**Analytes**	**Sorbent (amount)**	**Matrix (volume)**	**Extraction technique**	**Separation and detection technique**	**Recovery % (RSD %)**	**LODs**	**Comments**	**Reference**
DIBP, **DBP**, **DEHA,** DIOP	MOF-70 (17 mg)	Mineral water, carbonated soft drink, injection serum and sterile distilled water (5 mL)	µdSPE	GC-FID	40-68% (2-5%)	880-1040 ng/L	2-propanol was used as the desorption solvent. The final supernatant collected at µ-dSPE was used as a disperser solvent in the next DLLME procedure.	Pezhhanfar et al., *J. Food Compos. Anal.* **2021**, *104*, 104174.
DMP, DEP, DIBP, **DBP**, DMEP, BMPP, DEEP, **DNPP**, **DHP**, **BBP**, DBEP, **DCHP**, **DEHP**, **DNOP,** DNP	Graphene (3 mL, 1.5 mg/mL)	River and sea water (20 mL)	dSPE	GC-MS	71-117% (1-10%)	2000-6000 ng/L	TPP was used as IS. Colloidal graphene at a concentration of 1.5 mg/mL was prepared via chemical reduction of exfoliated GO. EtOAc was used as desorption solvent.	Wu et al., *Sci. Total Environ.* **2013**, *444*, 224-230.
**DEHP**	GO-MIPs (20 mg)	River, lake and rain water (600 mL)	µdSPE	HPLC-UV	82-92% (< 6.7%)	920 ng/L	Acetone was used as elution solvent.	Cheng et al., *J. Chromatogr. A* **2017**, *1511*, 85-91.
**BBP**, DBEP, **DIPP**, **DNPP**, **DEHA**, **DCHP**, **DEHP**, **DNOP,** **DINP**	Basolite® F300 (120 mg)	Tap, pond and waste water (50 mL)	dSPE	HPLC-MS	70-118% (1-19%)	6.6-20.7 ng/L	DHP-d_4 was used as IS. ACN was used as elution solvent. A small signal	González-Sálamo et al., *Talanta* **2019**, *195*, 236-244.

							enhancement was found for DEHP and DEHA in some matrices.	
DMP, DEP, **DBP**, **BBP**, **DEHP**, **DNOP**, 4-NP, 4-OP, 4-tOP, BPA, E1, E2, E3, EE2	C_{18} (80 mg)	Bottled mineral water (100 mL)	µdSPE	LC-MS	56-125% (0-22%)	1.6-23.2 ng/L	Five different sorbents were tested. MeOH was used as the extraction solvent.	Vivas et al., *Food Chem.* **2022**, *370*, 131062.
DBP, **DEHP**, DOA	TMU-6 (7 mg)	Bottled mineral water and boiling water kept in plastic containers (75 mL)	µdSPE	GC-FID	88-110% (4-8%)	200-700 ng/L	EtOAc was used as elution solvent.	Tahmasebi et al., *RSC Adv.* **2016**, *6*, 40211-40218.
DMP, DBP, **BBP**, **DEHP**	GO@LDH@SPAN (5 mg)	Drinking water and distilled herbal beverages (10 mL)	µdSPE	GC-MS	55-113% (0-10%)	60-300 ng/L	BnBzO was used as IS. Ace:MeOH (80:20, v/v) was used as elution solvent. The sorbent could be used for at least 4 times.	Otoukesh et al., *J. Chromatogr. A* **2020**, *1625*, 461307.
DMP, DEP, DBP **BBP**, **DEHP**, **DNOP**	PEMATrp DMIM (60 mg)	Plastic bottled water and artificial saliva (200 mL)	µdSPE	GC-MS	- (-)	310-410 ng/L	BnBzO was used as IS. DCM was used as elution solvent.	Özer et al., *J. Chromatogr. A* **2017**, *1500*, 53-60.
**DIPP, DNPP, DHP, BBP, DEHA, DCHP, DEHP, DNOP, DINP, DIDP**	**Chain-shattering polymer (30 mg)**	**Tap, waste and spring water**	**µdSPE**	**GC-MS**	**63-132% (1-16%)**	**2.03-41.6 ng/L**	**Four ISs were used. EtOAc was used as elution solvent.**	**This work**

μdSPE: micro dispersive solid-phase extraction; 4-NP: 4-nonylphenol; 4-OP:4- octylphenol; 4-tOP: 4-tert-octylphenol; Ace: acetone; ACN: acetonitrile; BBP: benzyl butyl phthalate; BMPP: bis(4-methyl-2-pentyl) phthalate; BnBzO: benzyl benzoate; BPA: bisphenol A; DBEP: bis(2-butoxyethyl) phthalate; DBP: dibutyl phthalate; DCHP: dicyclohexyl phthalate; DCM: dichloromethane; DEEP: bis(2-ethoxyethyl) phthalate; DEHA: di(2-ethylhexyl) adipate; DEHP: di(2-ethylhexyl) phthalate; DEP: diethyl phthalate; DHP: dihexyl phthalate; DHP-d_4: dihexyl phthalate-3,4,5,6-d_4; DIBP: diisobutyl phthalate; DIDP: diisodecyl phthalate; DINP: diisononyl phthalate; DIOP: diisooctyl phthalate; DIPP: diisopentyl phthalate; DLLME: dispersive liquid-liquid microextraction; DMEP: bis(2-methoxyethyl) phthalate; DMIM: dummy molecularly imprinted microbead; DMP: dimethyl phthalate; DNOP: di-*n*-octyl phthalate; DNP: dinonyl phthalate; DNPP: di-n-pentyl phthalate; DOA: dioctyladipate; dSPE: dispersive solid-phase extraction; E1: estrone; E2: 17β-estradiol; E3: estriol; EE2: 17α-ethinylestradiol; EtOAc: ethyl acetate; FID: flame ionization detection; GC: gas chromatography; GO: graphene oxide; HPLC: high-performance liquid chromatography; IS: internal standard; LC: liquid chromatography; LDH: layered double hydroxide; LOD: limit of detection; MeOH: methanol; MIP: molecularly imprinted polymer; MOF: metal organic framework; MS: mass spectrometry; PEMATrp: poly(ethylene glycol dimethacrylate N-methacryloyl-L-tryptophan methyl ester); RSD: relative standard deviation; SPAN: sulfonated polyaniline: TPP: triphenyl phosphate; UV: ultraviolet.

POLYMER SYNTHESIS

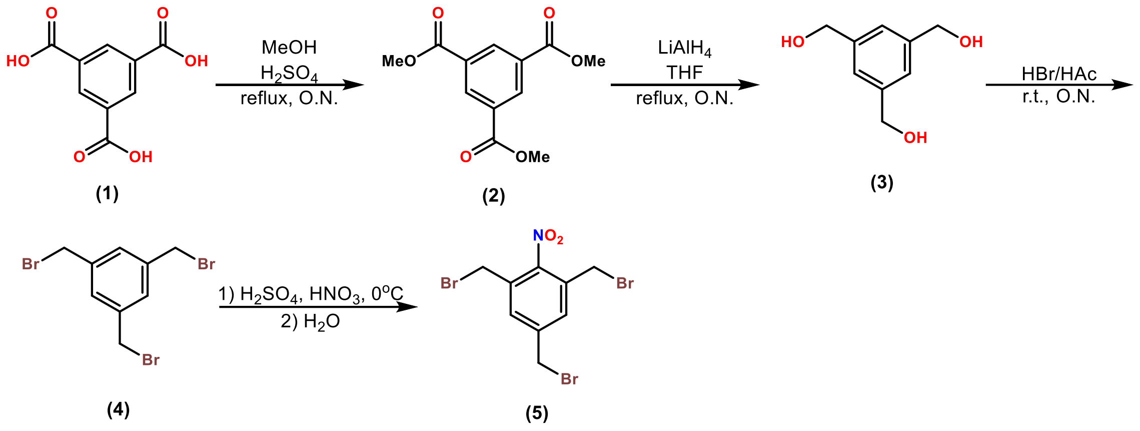

Compound (2): trimethyl benzene-1,3,5-tricarboxylate
Trimesic acid (**1**) (3.00 g, 14.3 mmol) was dissolved in 60 mL of MeOH. H_2SO_4 was slowly added (0.75 mL) and the reaction was refluxed for 24 h before being allowed to cool to r.t. An aqueous $NaHCO_3$ solution (saturated) was slowly added to neutralize the mixture. The aqueous phase was extracted with Et_2O (3x20 mL), dried over $MgSO_4$, filtered and concentrated under reduced pressure. Compound **2** was obtained as a white solid[8] in quantitative yield.

^{1}H NMR (500 MHz, $CDCl_3$, 298 K) δ (ppm): 8.86 (s, 3H), 3.98 ppm (s, 9H).
^{13}C NMR (150 MHz, $CDCl_3$, 298 K) δ (ppm): 165.58, 134.75, 131.37, 52.78.
HRMS (EI-TOF): m/z: calc for $C_{12}H_{12}O_6$ [M+]: 252.0634, found: 252.0639.

Compound (4): 1,3,5-tris(bromomethyl)benzene
According to the literature,[8] triester **2** (3.6 g, 14.3 mmol) was dissolved in THF (75 mL) and it was added dropwise over a solution of $LiAlH_4$ (2.56 g, 64.4 mmol) in THF at 0 °C. The reaction was refluxed during 24 h before being allowed to cool. Upon cooling, H_2O was carefully added (50 mL) and the mixture was filtered through a celite pad, and the filter cake was washed with DCM. The volatiles were concentrated under reduced pressure. After that, the crude of alcohol **3** was mixed with 20.9 mL of HBr in HAc solution (33% w/v) and stirred over night to obtain a needle-like off-white solid that was filtered, generously washed with water and dried under reduced pressure. [9] 4.03 g, 79% yield.

^{1}H NMR (500 MHz, $CDCl_3$, 298 K) δ (ppm): 7.34 (s, 3H), 4.46 ppm (s, 6H).
^{13}C NMR (150 MHz, $CDCl_3$, 298 K) δ (ppm): 139.21, 129.72, 32.31.
HRMS (EI-TOF): m/z: calc for $C_9H_9{}^{81}Br_3$ [M+]: 359.8193, found: 359.8217; calc for $C_9H_9{}^{79}Br_3$ [M+]: 353.8254, found: 353.8262

Compound (5): 1,3,5-tris(bromomethyl)-2-nitrobenzene
A mixture of HNO_3 (27 mL) and H_2SO_4 (31 mL) in a round bottom flask was cooled down in an ice-water bath. Compound **4** (11.5 g, 32.2 mmol) was carefully added in a period of 10 min keeping the temperature at 0 °C for 1 h after the complete addition. The mixture was allowed to reach room temperature and an ice-water mixture was added. The aqueous phase was extracted with DCM (3x50 mL), and the organic layer was washed with H_2O (3x40 mL), dried over $MgSO_4$, filtered and concentrated under reduce pressure. Compound **5** was obtained as a white solid after purification by column chromatography on silica gel (AcOEt/Hex 5:95). 9.83 g, 76% yield.

^{1}H NMR (500 MHz, $CDCl_3$, 298 K) δ (ppm): 7.52 (s, 2H), 4.48 (s, 4H), 4.45 ppm (s, 2H).
^{13}C NMR (150 MHz, $CDCl_3$, 298 K) δ (ppm): 141.75, 132.28, 131.8, 30.33, 26.27.
HRMS (EI-TOF): m/z: calc for $C_9H_8NO_2{}^{81}Br_3$ [M+]: 404.8044, found: 404.8058; calc for $C_9H_8NO_2{}^{79}Br_3$ [M+]: 398.8105, found: 398.8110

Polymer Synthesis:

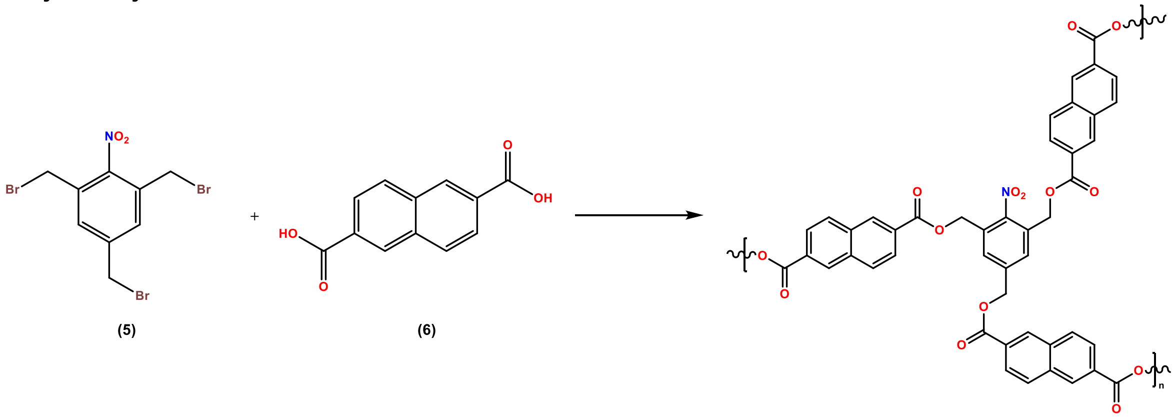

NO_2-polymer: compound **5** (0.84 g, 3.9 mmol), K_2CO_3 (1.25 g, 9.1 mmol) and commercially available **6** (1.04 g, 2.6 mmol) were placed in a round bottom flask under N_2 atmosphere with a magnetic stirrer. Dry DMF (13 mL, 0.2M) was added and the solution was heated for 18 h at 80 ^{o}C. The mixture was cooled to room temperature and 20 mL of Milli-Q H_2O were added appearing a white solid. The mixture was let to stir for 1 additional hour and the solid was filtered, washed with Milli-Q H_2O (2x20 mL), MeOH HPLC grade (2x15 mL) and ACN HPLC grade (2x15 mL). Finally, the white solid was dried in high vacuum. 1.17 g, 93% yield.

Before use, if necessary, the polymer was washed with an appropriate volume of different organic solvents (i.e., ACN, MeOH or AcOEt) to remove any impurities or plasticizers. Then, it was dried under vacuum and acidified with a 0.1 M HCl solution and subsequently washed with Milli-Q water until neutral pH. Finally, it was completely dried under vacuum.

DEGRADATION STUDIES

Photodegradation:

1.4 mg of the polymer (2.89 mmol) was added to a quartz NMR tube, followed by 0.17 mL of a 0.0145 M solution of hexamethyldisilane in DMSO-D_6 (as IS). The volume of the tube was adjusted to 0.7 mL and exposed to UV light with an ACE-Hanovia photochemical lamp 7830-60 (450W) and monitored by ^{1}H-NMR experiment.

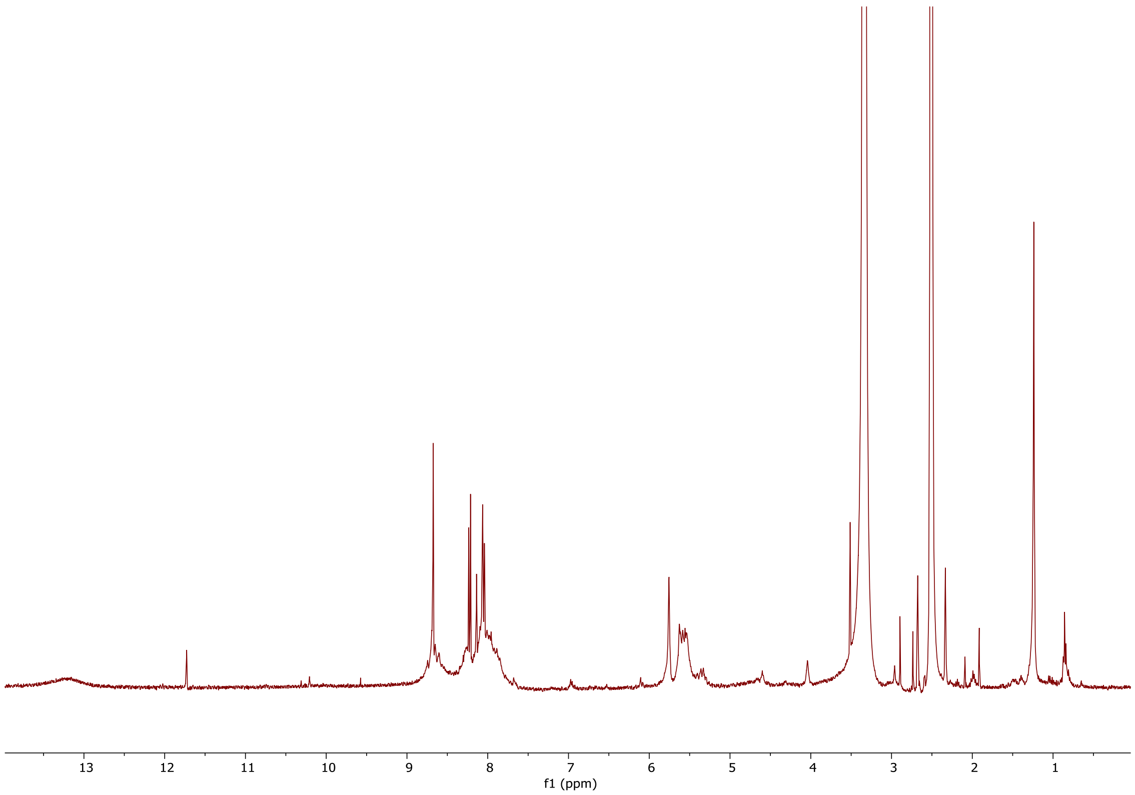

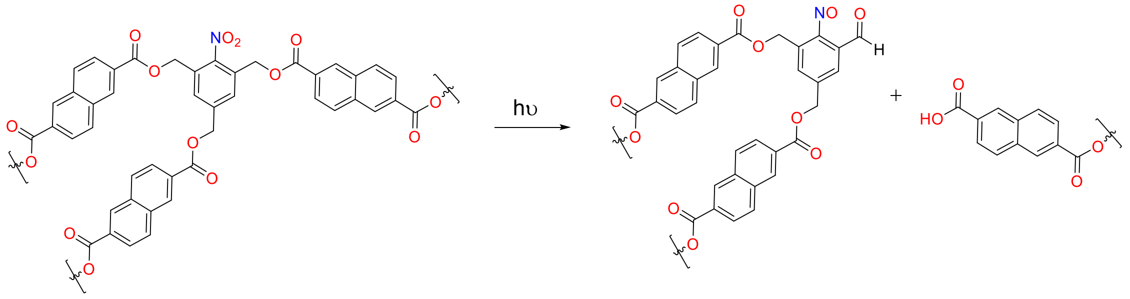

To obtain the concentration of the carboxylic acid, 1 peak of IS and 1 signal of the acid were deconvolutionated to obtain the areas. With those values, a quotient of both was done to obtain the concentration.

IS: Internal Standard

$$0.0145\ \text{M of IS} = \frac{\text{x mmol}}{0.17\ \text{mL}} \rightarrow 0.00246\ \text{mmol} \xrightarrow{0.7\ \text{mL}} 0.0035\ \text{M}\ \ of\ IS$$

$$\text{Acid (M)} = \frac{\text{Area of acid}}{\left(\frac{\text{Area of IS}}{\text{Conc IS}}\right)}$$

Time (min)	Area of IS	Area of acid	Conc (M)
30	$4.819{\cdot}10^{7}$	$2.286{\cdot}10^{5}$	$1.67{\cdot}10^{-5}$
60	$5.628{\cdot}10^{12}$	$6.575{\cdot}10^{10}$	$4.11{\cdot}10^{-5}$
90	$5.942{\cdot}10^{12}$	$1.166{\cdot}10^{11}$	$6.91{\cdot}10^{-5}$
120	$5.339{\cdot}10^{12}$	$1.359{\cdot}10^{11}$	$8.97{\cdot}10^{-5}$
150	$4.545{\cdot}10^{12}$	$1.494{\cdot}10^{11}$	$1.16{\cdot}10^{-4}$
210	$5.471{\cdot}10^{12}$	$2.113{\cdot}10^{11}$	$1.36{\cdot}10^{-4}$
255	$4.211{\cdot}10^{12}$	$1.622{\cdot}10^{11}$	$1.36{\cdot}10^{-4}$
355	$4.077{\cdot}10^{12}$	$1.577{\cdot}10^{11}$	$1.36{\cdot}10^{-4}$

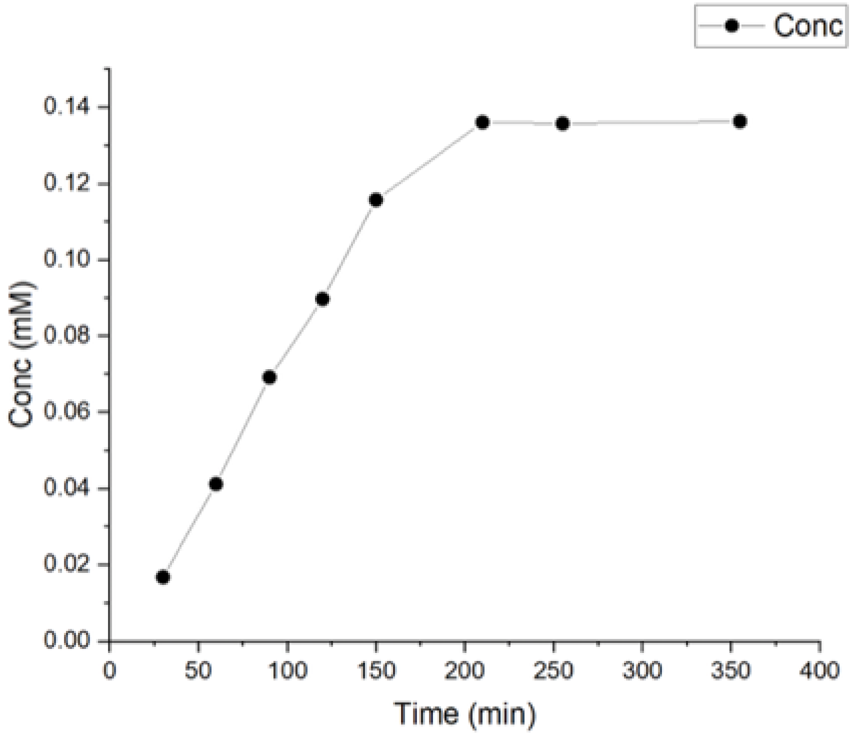

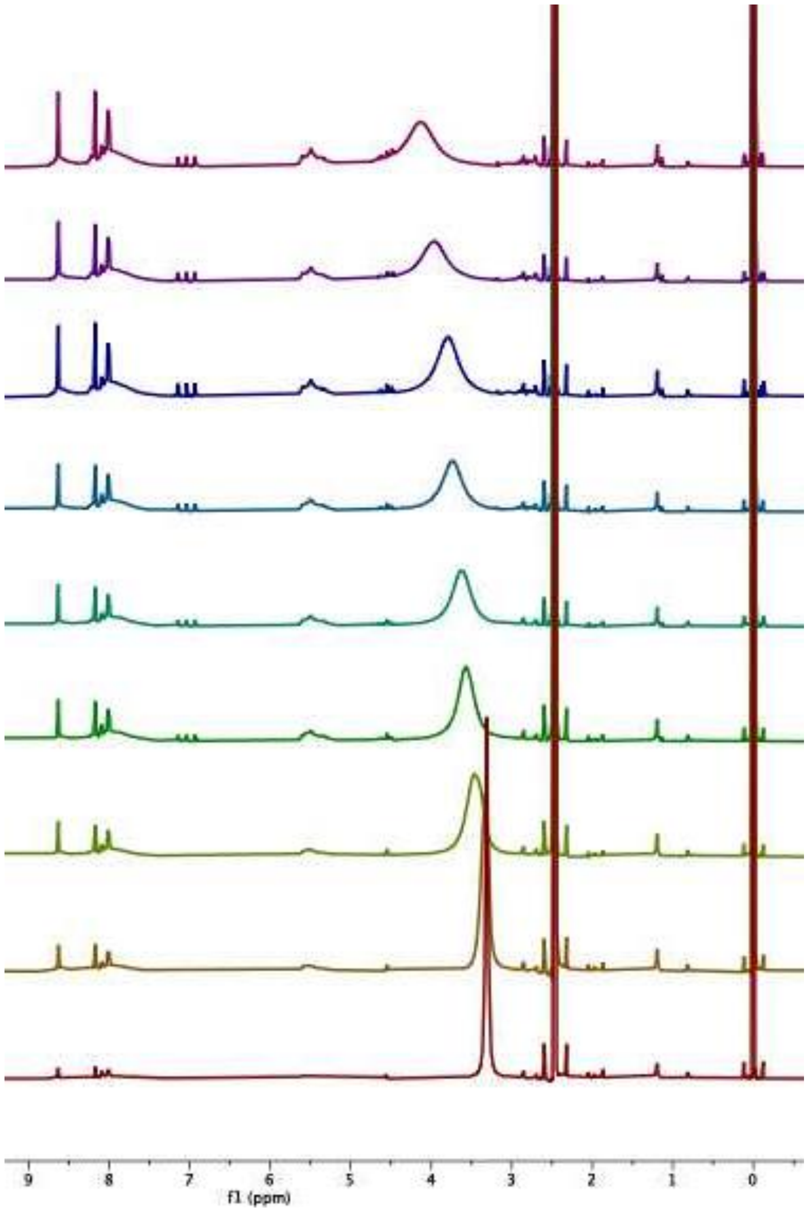

Self-immolation:

1.2 mg of the polymer (2,48 mmol) was added to a NMR tube, followed by 0.17 mL of a 0.0145 M solution of hexamethyldisilane in DMSO-D_6 (as internal standard). The volume of the tube was adjusted to 0.7 mL by adding DMSO-D_6 (0,33 mL) and a saturated D_2O solution of $Na_2S_2O_4$ (0.2 mL) and heated to 100 ºC in an oil bath. The process was monitored by ^{1}H-NMR experiment.

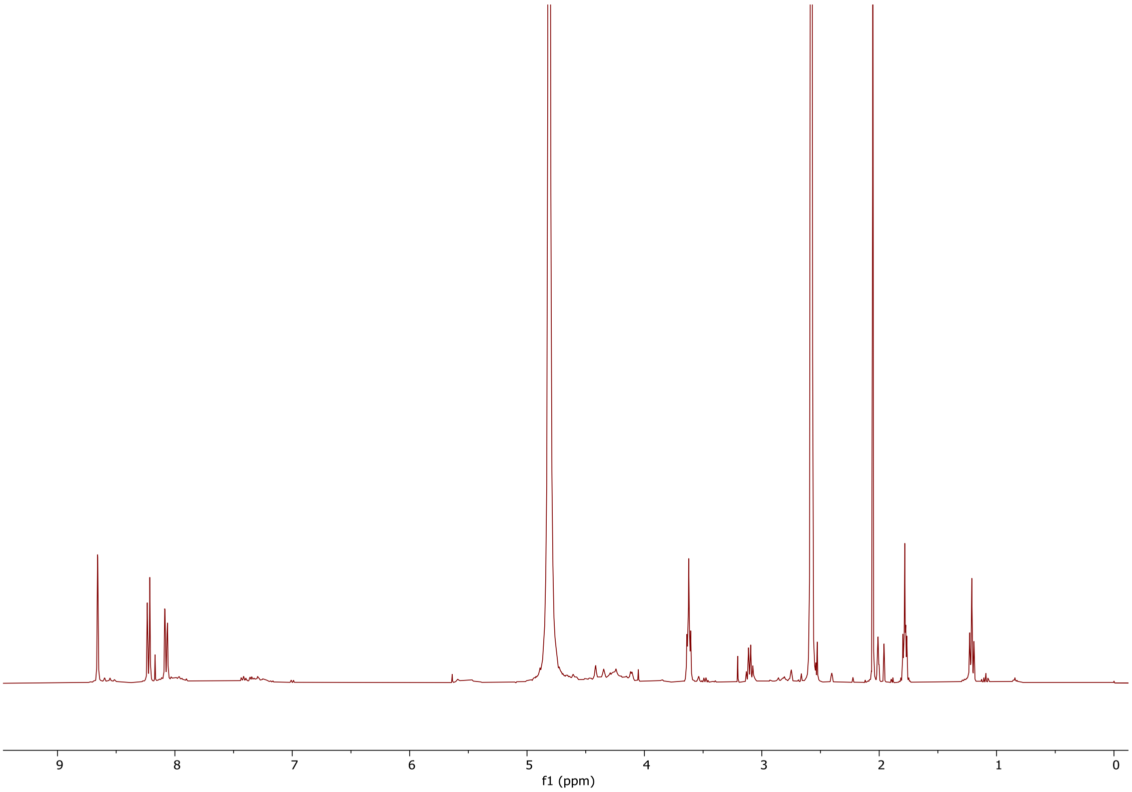

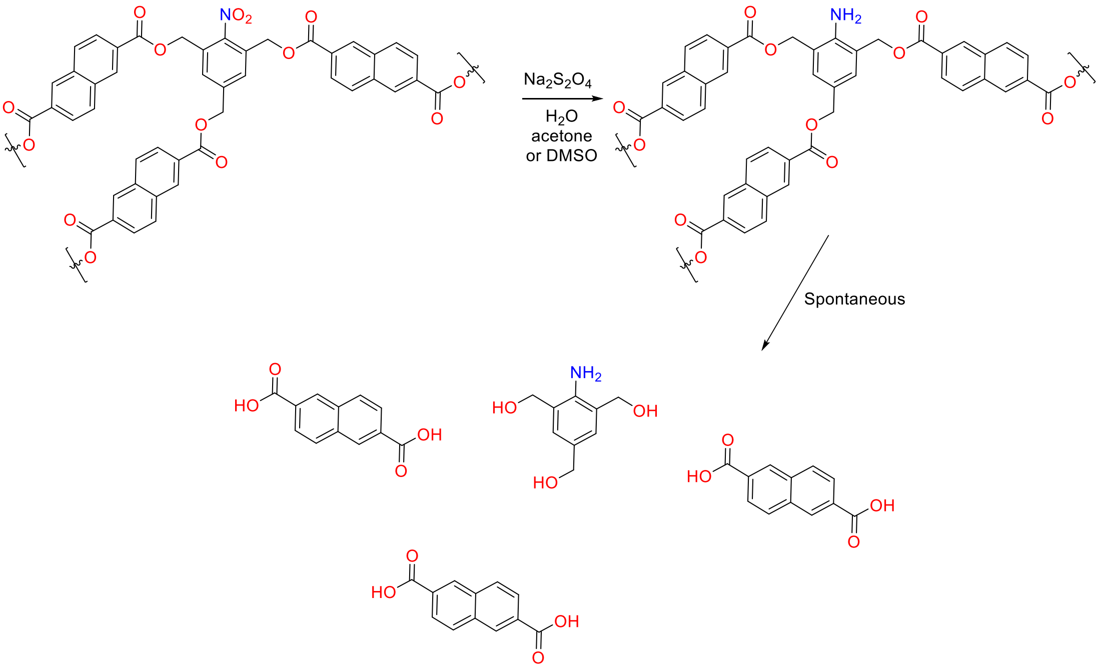

Self-immolation NMR is cleaner than the photodegradation because the latter only break one of the three links to the central node, and therefore several soluble oligomers are expected, which yields a messier spectrum with wide signals combined with sharp ones. Self-immolation however, leads to a theoretically complete degradation.

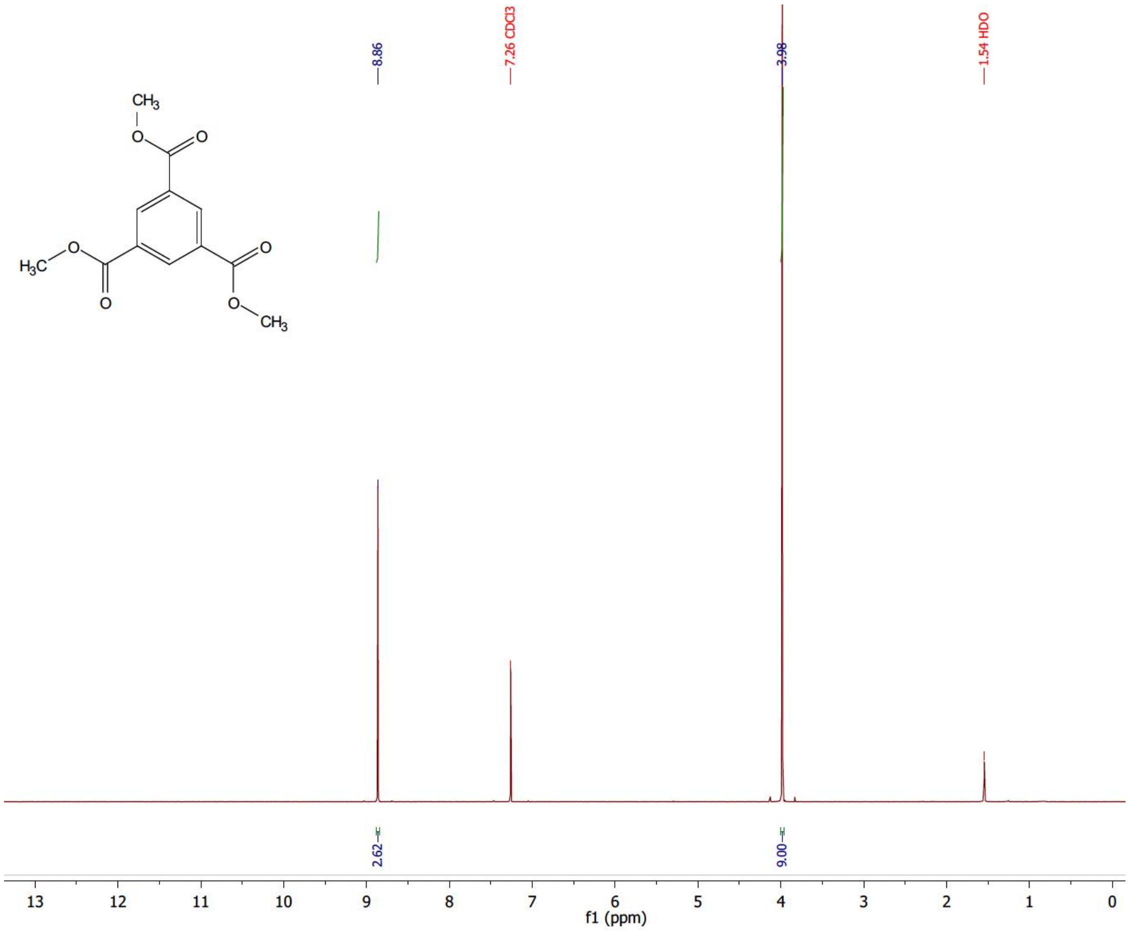
8.86
7.26 CDCl3
3.98
1.54 HDO
2.62
9.00
f1 (ppm)
CH3
O
H3C

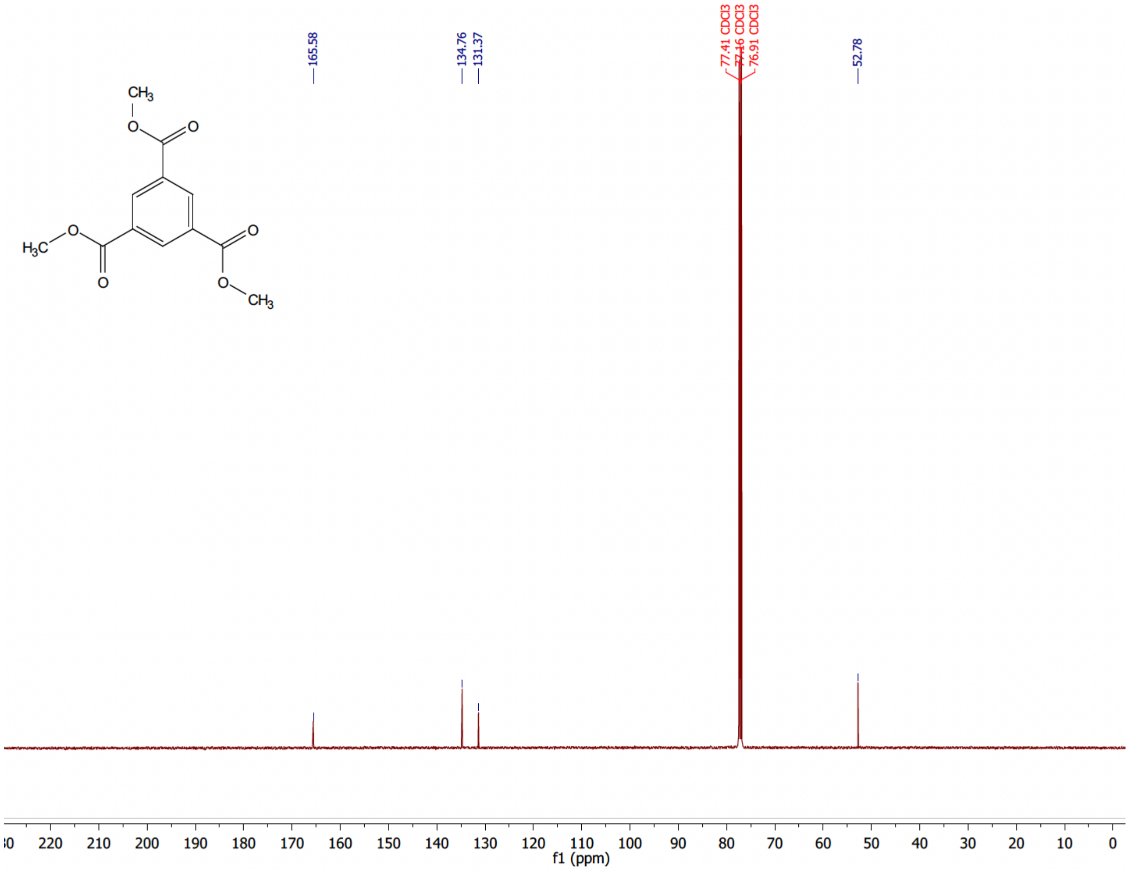
165.58
134.76
131.37
77.41 CDCl3
77.16 CDCl3
76.91 CDCl3
52.78
f1 (ppm)
CH3
O
H3C

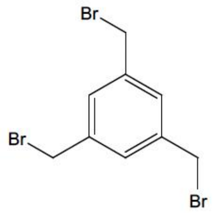
Br
Br
Br

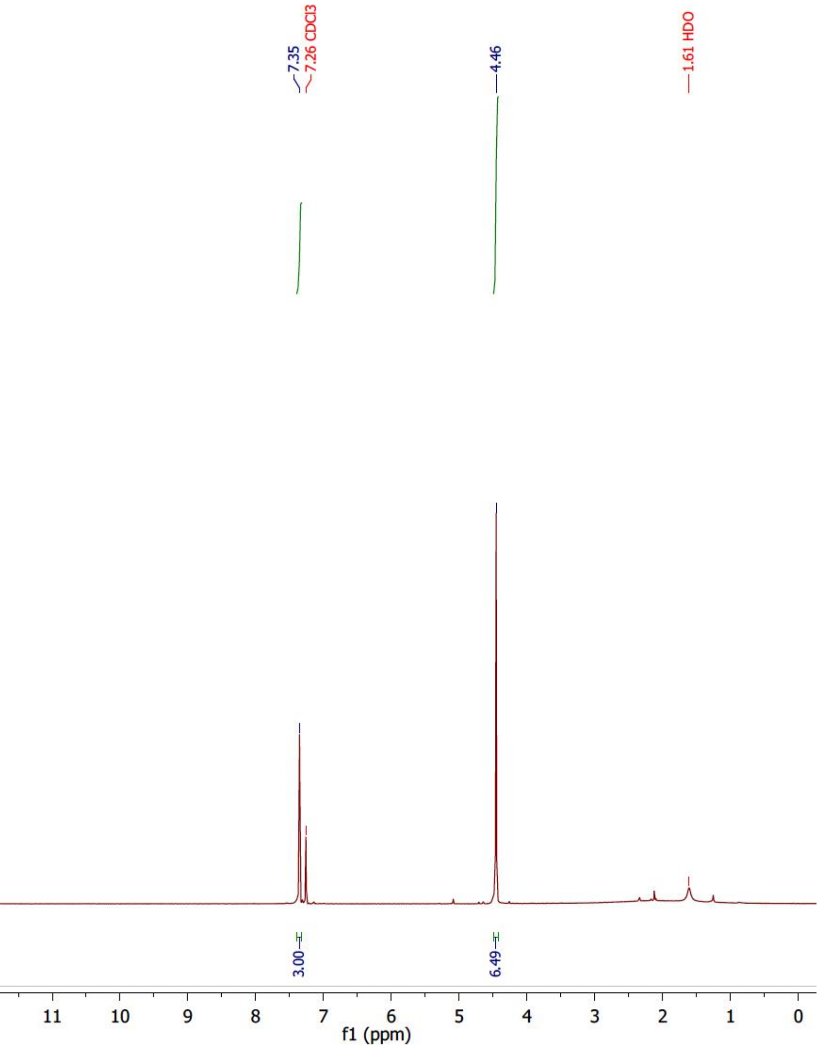
7.35
7.26 CDCl3
4.46
1.61 HDO
3.00
6.49
16 15 14 13 12 11 10 9 8 7 6 5 4 3 2 1 0
f1 (ppm)

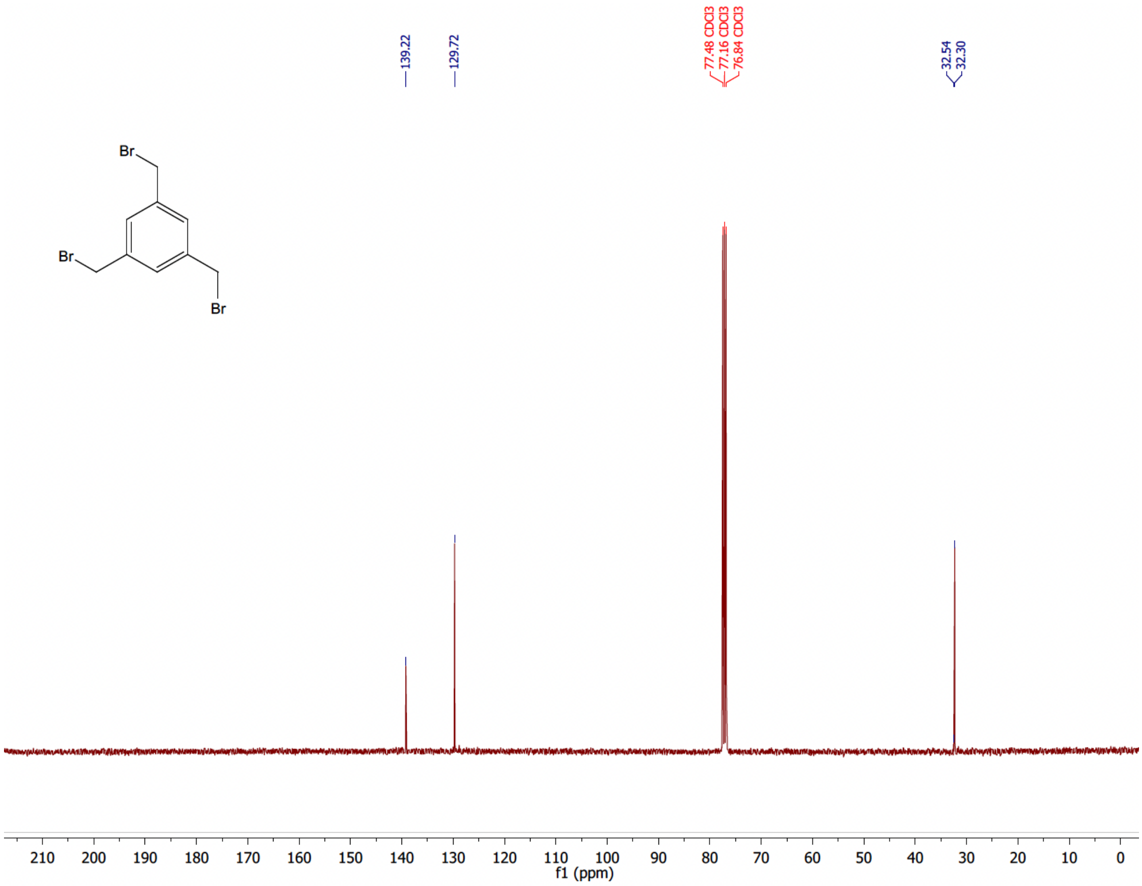
139.22
129.72
77.48 CDCl3
77.16 CDCl3
76.84 CDCl3
32.54
32.30
Br
Br
Br
210 200 190 180 170 160 150 140 130 120 110 100 90 80 70 60 50 40 30 20 10 0
f1 (ppm)

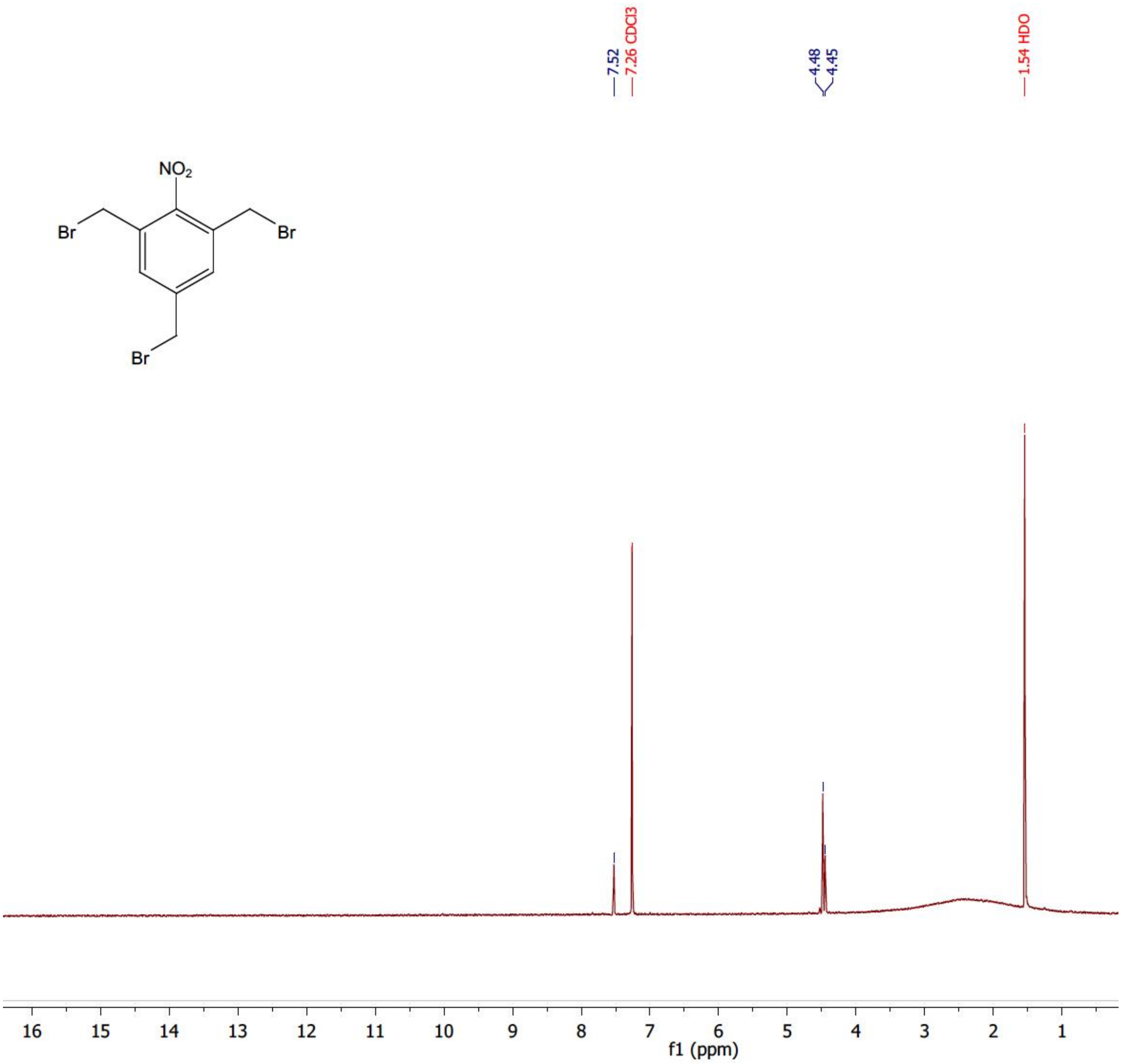
7.52
7.26 CDCl3
4.48
4.45
1.54 HDO
NO2
Br
Br
Br
16
15
14
13
12
11
10
9
8
7
6
5
4
3
2
1
f1 (ppm)

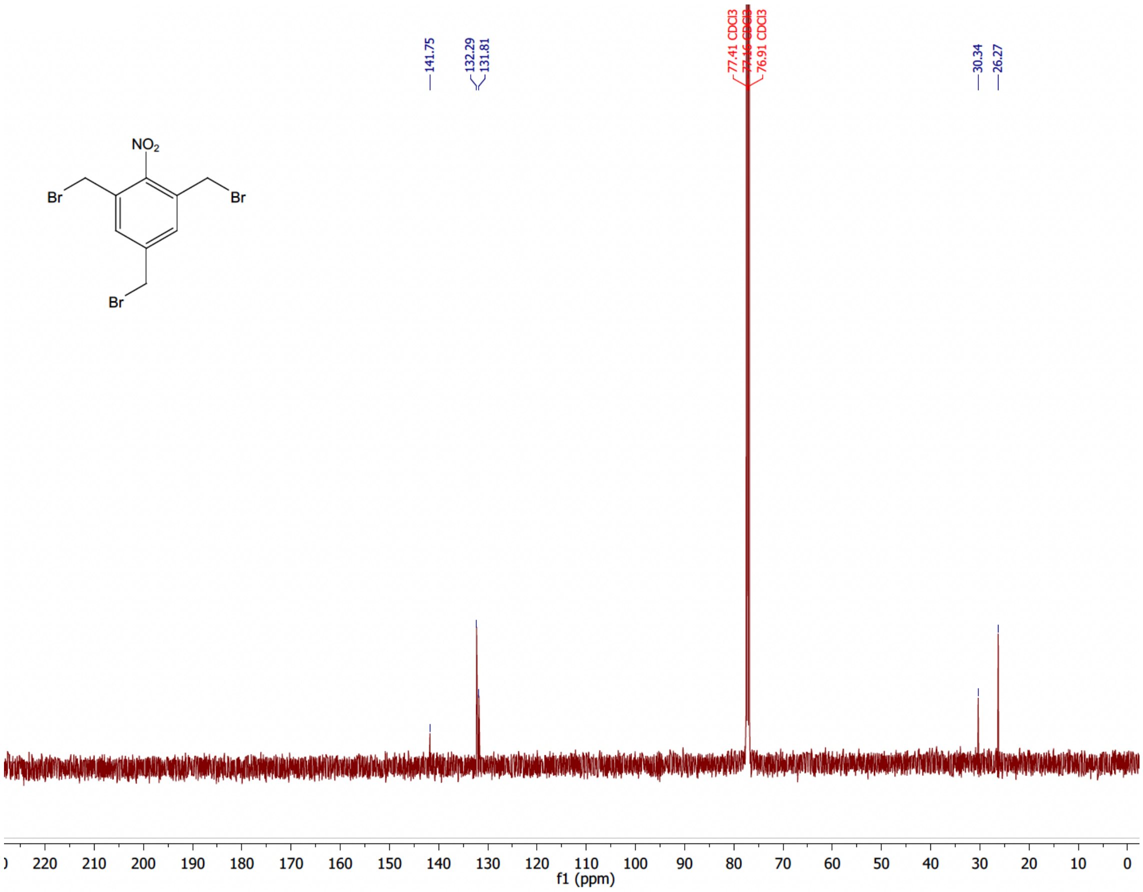

NO2
Br
Br
Br
141.75
132.29
131.81
77.41 CDCl3
77.16 CDCl3
76.91 CDCl3
30.34
26.27
220 210 200 190 180 170 160 150 140 130 120 110 100 90 80 70 60 50 40 30 20 10 0
f1 (ppm)

DEGRADATION-REPOLYMERIZATION CYCLE STUDY

A reductive degradation with sodium dithionite (335 mg) starting from 100 mg of polymer **CSP-1** in 20 mL of acetone:water (9:1, v/v) was carried out. After 12 h of reflux, the polymer was degraded. The reaction was cooled down to room temperature, and the solvents were evaporated and the solid was vacuum dried. A portion of such crude residue was dissolved in DMF and 27 mg of 1,3,5-tris(bromomethyl)-2-nitrobenzene (TBMNB) and 32 mg of K_2CO_3 were added to the reaction flask and the reaction was performed as explained before.

A white polymer was obtained, which gives an IR spectrum that matched that of the original polymer, confirming that the crude mixture obtained after the degradation can be used for repolymerization of **CSP-1**.

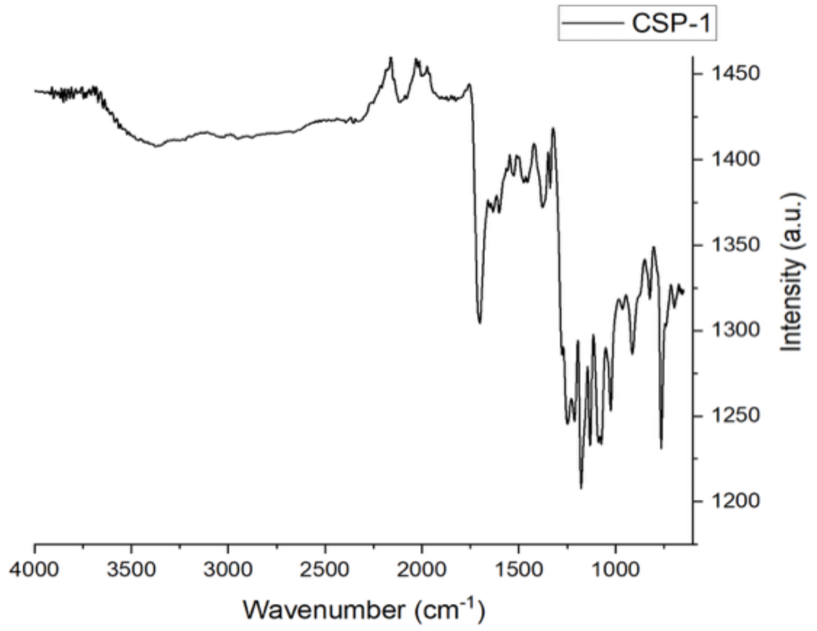

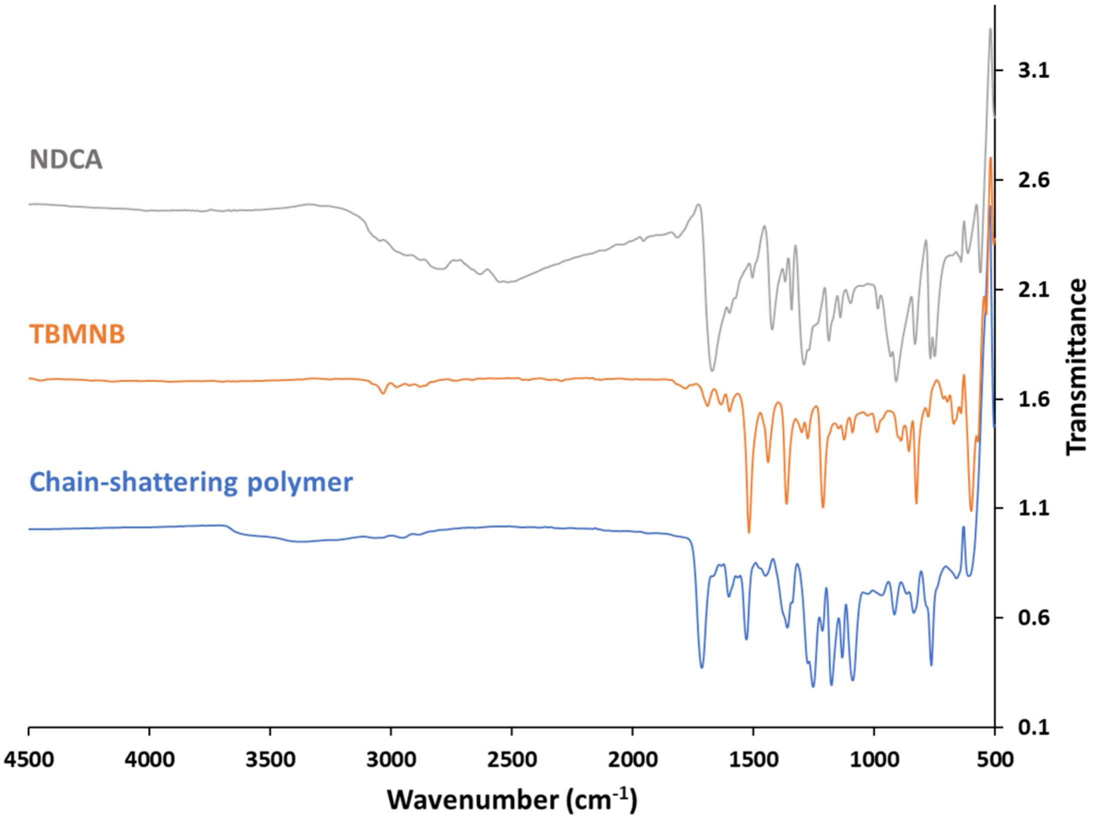

Figure S1.- FTIR spectra of NDCA, TBMNB and the CSP-1.

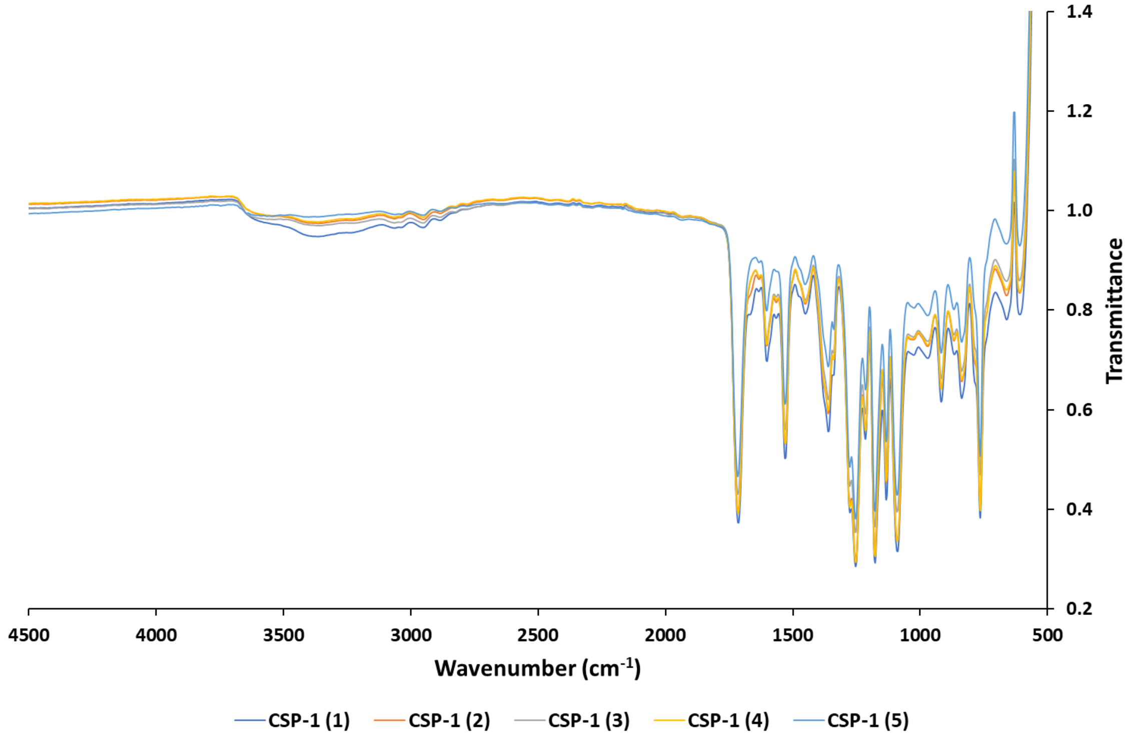

Figure S2.- FTIR spectra of the CSP-1 synthesized in different batches.

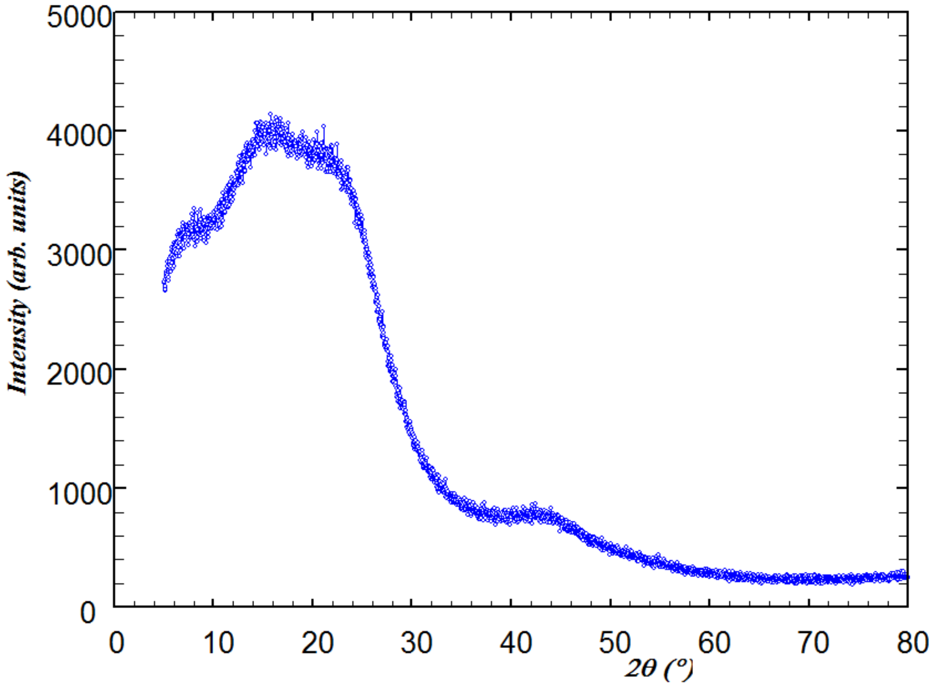

Figure S3.- X-ray diffraction pattern obtained for the polymer showing the shape of a predominantly amorphous material.

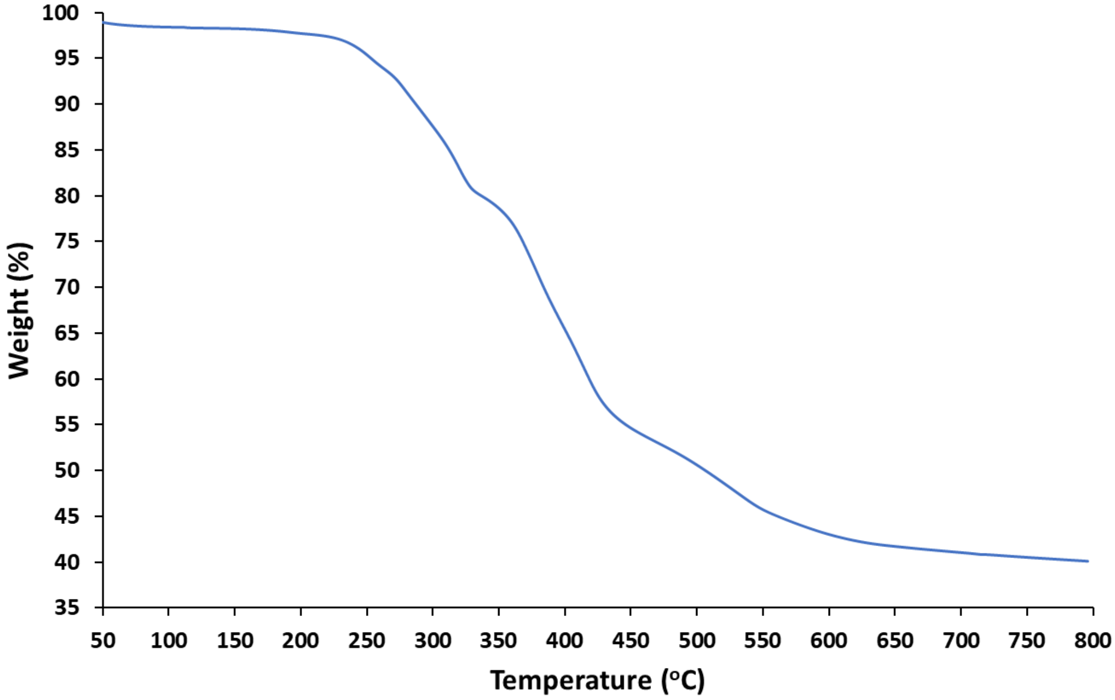

Figure S4.- TGA curve of the CSP-1.

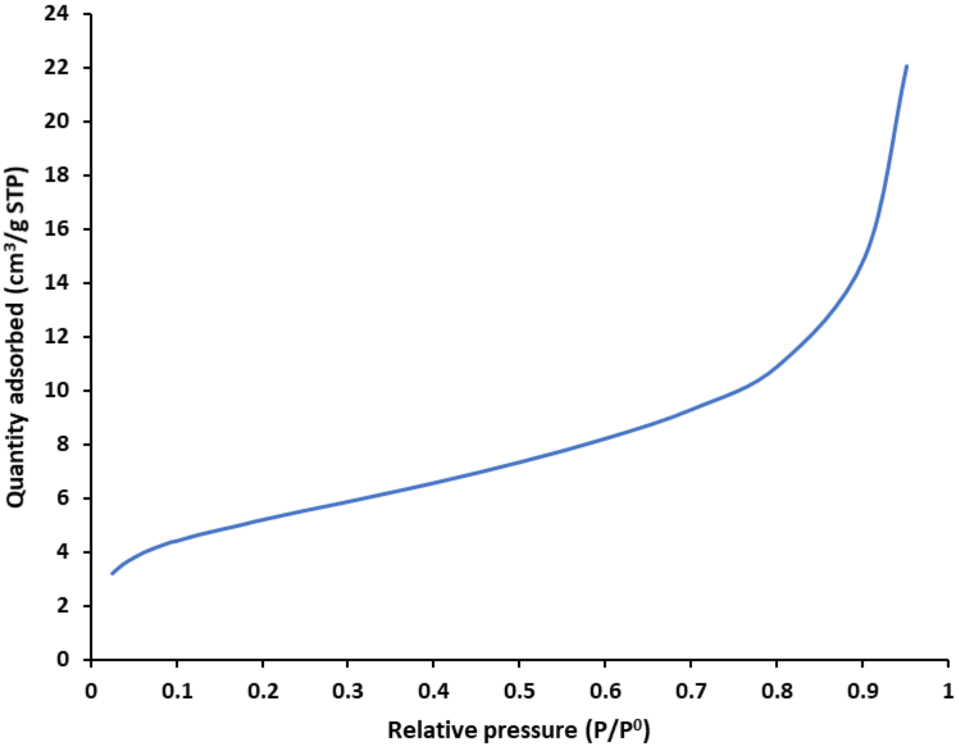

Figure S5.- N_2 adsorption isotherm plot of the CSP-1.

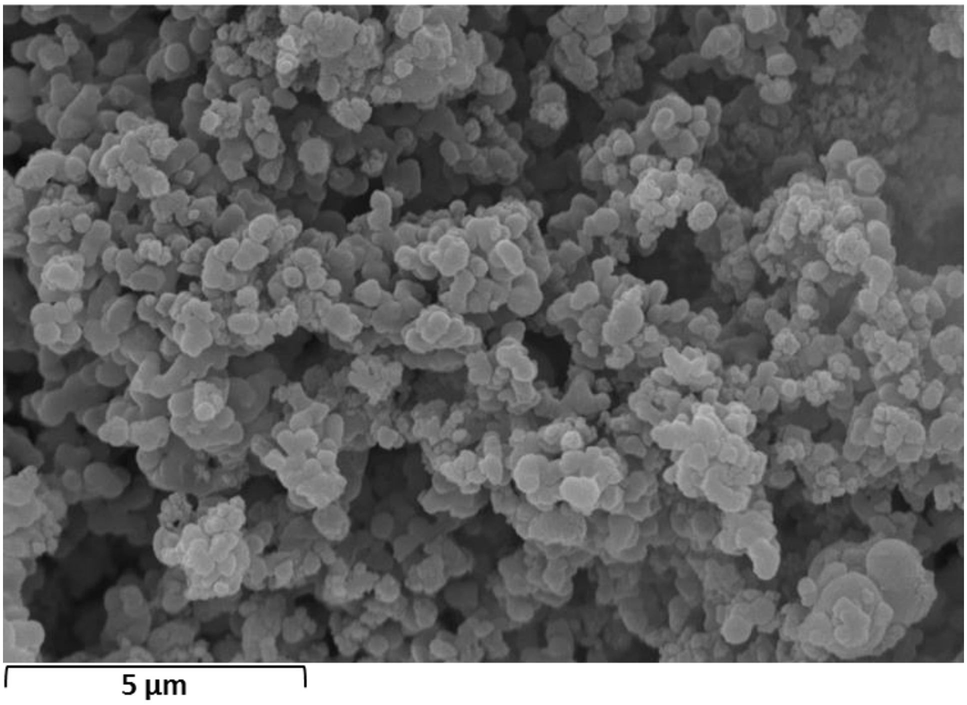

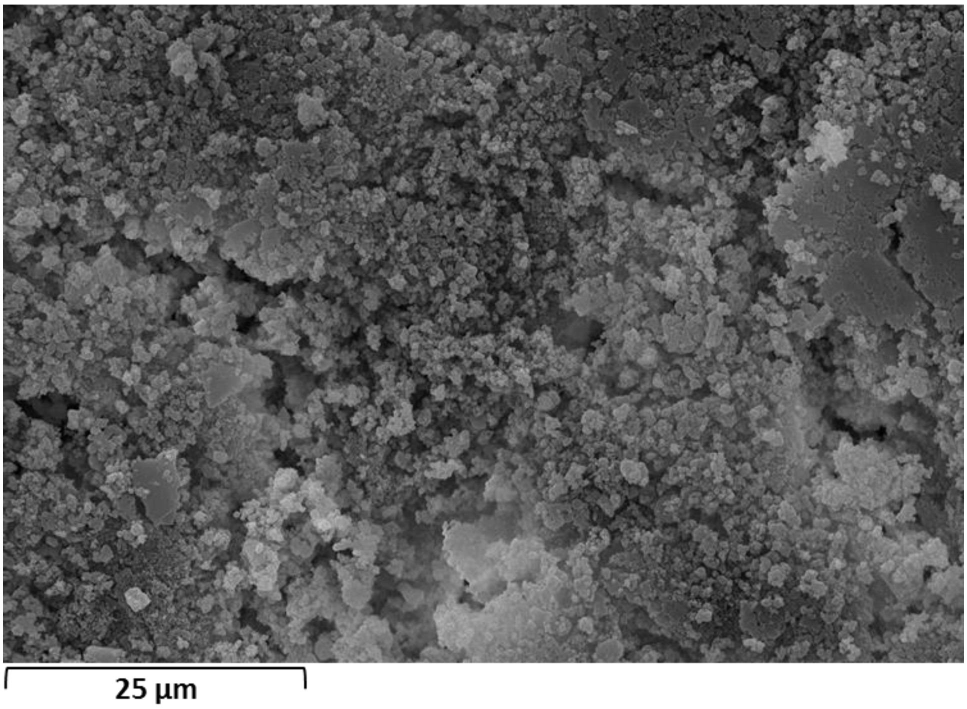

Figure S6.- SEM images of the CSP-1.

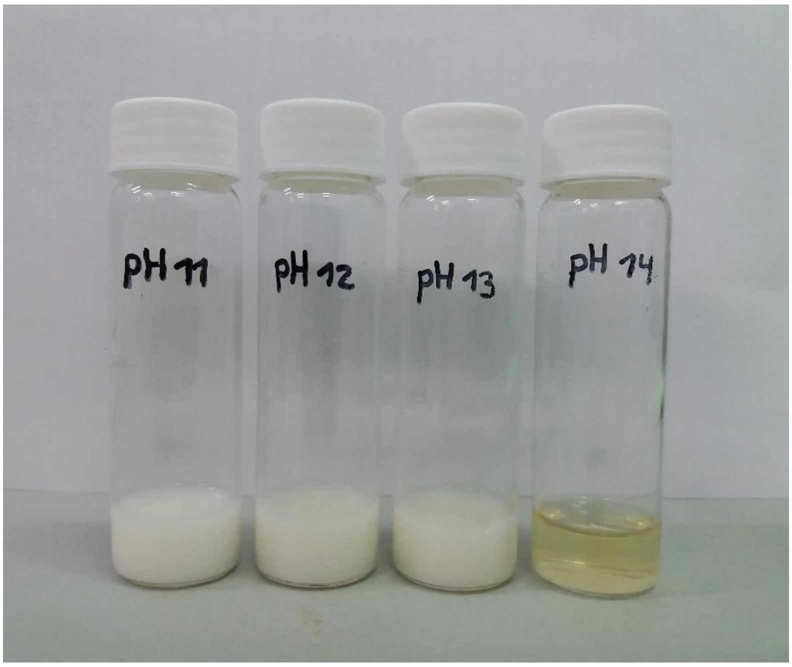

Figure S7.- Photograph of the resulting mixtures of 40 mg of the polymer and 5 mL of water at pH 11, 12, 13 and 14.

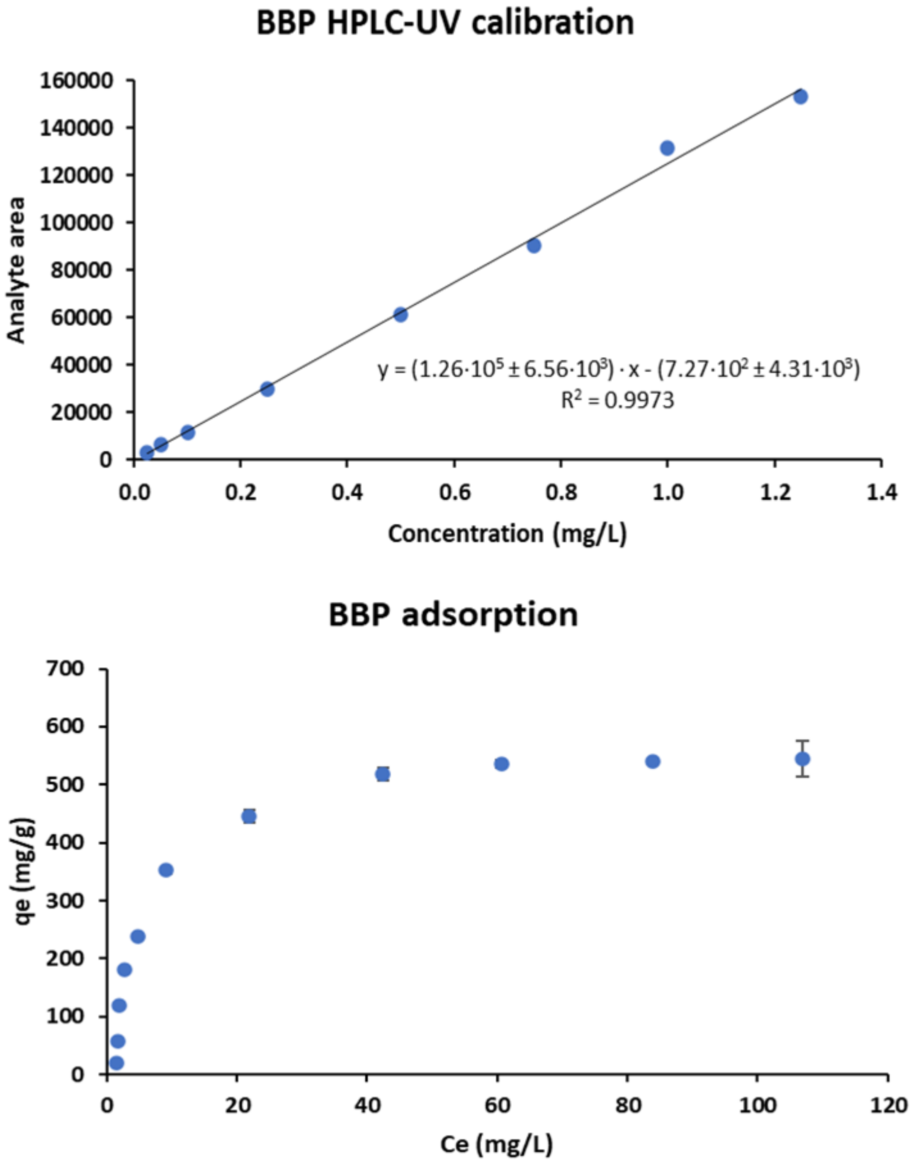

Figure S8.- BBP HPLC-UV calibration curve (top) and adsorption isotherm of BBP onto CSP-1 (down).

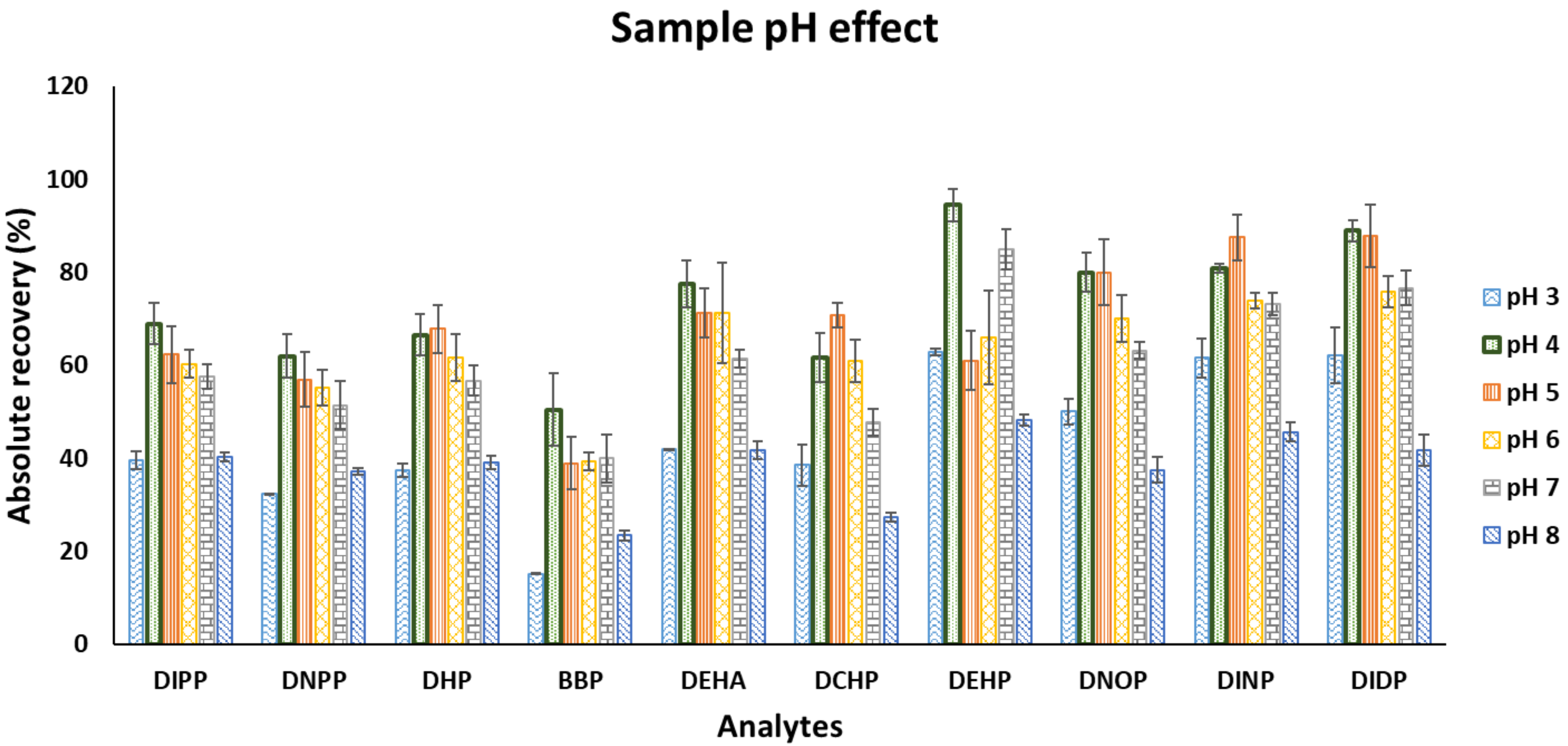

Figure S9.- Effect of the pH during the extraction step on the peak areas of the target PAEs. Extraction conditions: 30 mg sorbent, 50 mL of spiked Milli-Q water at 125 µg/L, manual agitated for 2.5 min and elution with 15 mL of ethyl acetate.

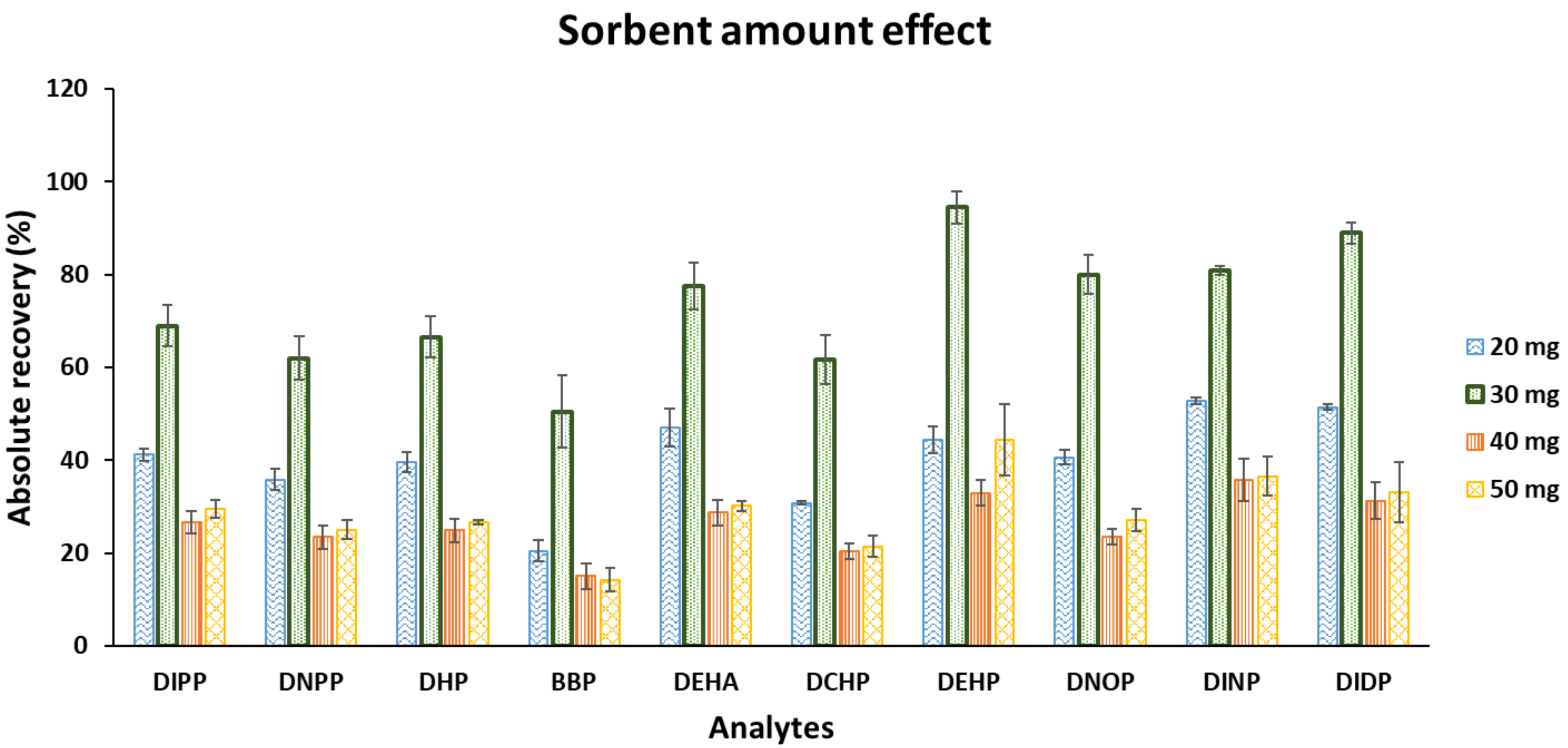

Figure S10.- Effect of the sorbent amount used in the extraction step on the peak areas of the selected PAEs. Extraction conditions: 50 mL of spiked Milli-Q water at 125 µg/L (pH 4.0), manual agitated for 2.5 min, and elution with 15 mL of ethyl acetate.

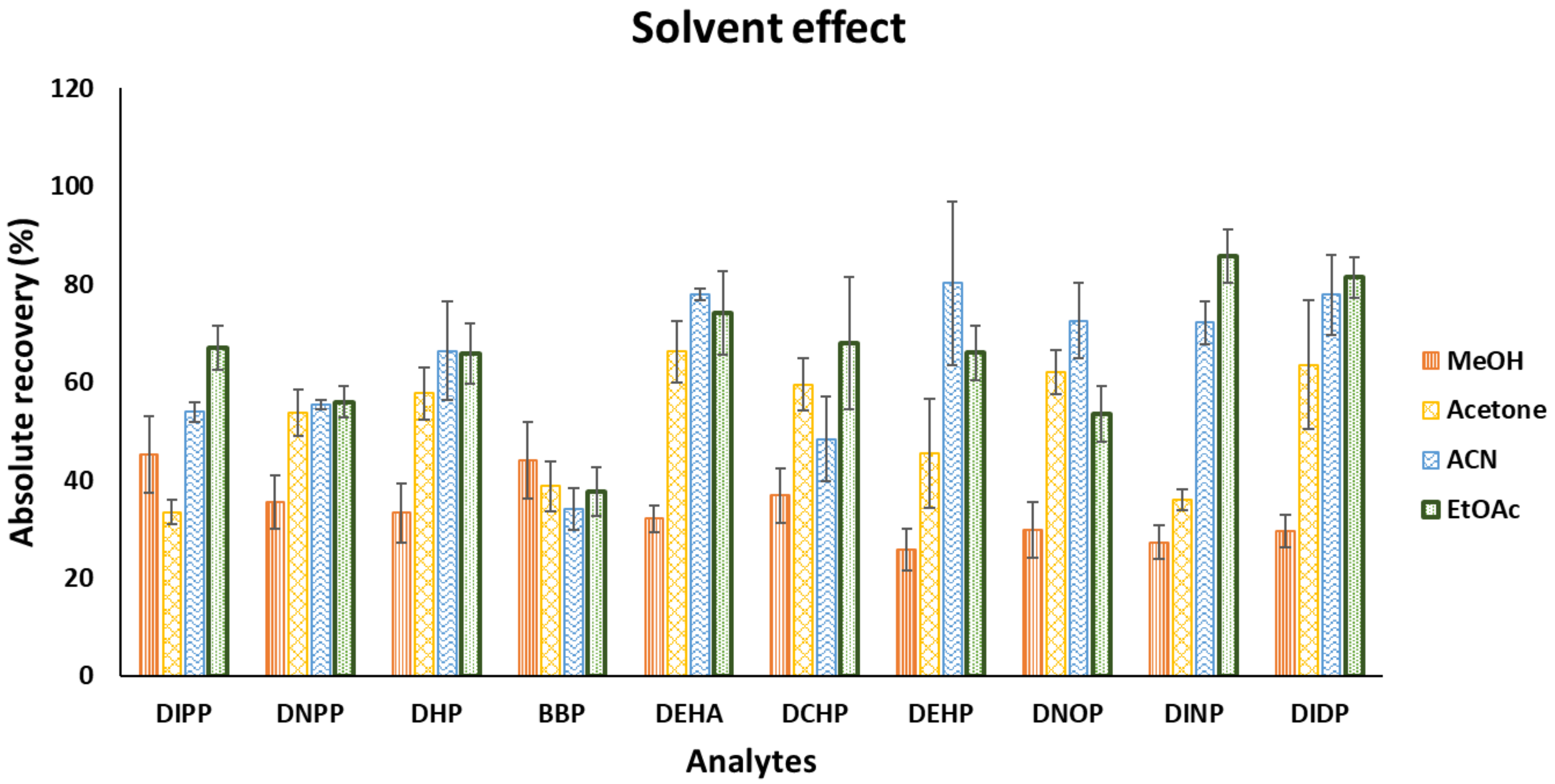

Figure S11.- Effect of the solvent used in the elution step on the peak areas of the selected analytes. Extraction conditions: 30 mg sorbent, 50 mL of spiked Milli-Q water at 125 µg/L (pH 6.0), manual agitated for 2.5 min, and elution with 15 mL of each solvent.
